# Climate warming enhances biodiversity and stability of grassland soil phosphorus-cycling microbial communities

**DOI:** 10.1093/ismejo/wraf118

**Published:** 2025-06-18

**Authors:** Zijian Wang, Il Han, Jangho Lee, Guangyu Li, Peisheng He, Mathew T Baldwin, Jenny Kao-Kniffin, Liyou Wu, Jizhong Zhou, April Z Gu

**Affiliations:** Department of Biological and Environmental Engineering, College of Agriculture and Life Sciences, Cornell University, Ithaca, NY 14853, United States; Center for Research on Programmable Plant Systems, Cornell University, Ithaca, NY 14853, United States; School of Civil and Environmental Engineering, College of Engineering, Cornell University, Ithaca, NY 14853, United States; School of Civil and Environmental Engineering, College of Engineering, Cornell University, Ithaca, NY 14853, United States; School of Civil and Environmental Engineering, College of Engineering, Cornell University, Ithaca, NY 14853, United States; School of Civil and Environmental Engineering, College of Engineering, Cornell University, Ithaca, NY 14853, United States; Center for Research on Programmable Plant Systems, Cornell University, Ithaca, NY 14853, United States; School of Civil and Environmental Engineering, College of Engineering, Cornell University, Ithaca, NY 14853, United States; School of Integrative Plant Science, Cornell University, Ithaca, NY 14853, United States; Institute for Environmental Genomics, University of Oklahoma, Norman, OK 73019, United States; Department of Microbiology and Plant Biology, University of Oklahoma, Norman, OK 73019, United States; Institute for Environmental Genomics, University of Oklahoma, Norman, OK 73019, United States; Department of Microbiology and Plant Biology, University of Oklahoma, Norman, OK 73019, United States; School of Civil Engineering and Environmental Sciences, University of Oklahoma, OK 73019, United States; Earth and Environmental Sciences, Lawrence Berkeley National Laboratory, Berkeley, CA 94720, United States; Center for Research on Programmable Plant Systems, Cornell University, Ithaca, NY 14853, United States; School of Civil and Environmental Engineering, College of Engineering, Cornell University, Ithaca, NY 14853, United States

**Keywords:** climate change, soil phosphorus cycling, polyphosphate accumulating organisms, microbial biodiversity, sustainable phosphorus management, single-cell Raman spectroscopy, fluorescence-activated cell sorting

## Abstract

Climate warming poses significant challenges to global phosphorus sustainability, an essential component of Earth biogeochemistry cycling and water-food-energy nexus. Despite the crucial role of polyphosphate-accumulating organism as key functional microbial agents in phosphorus cycling, the impacts of global climate warming on polyphosphate accumulating organism communities remain largely enigmatic. This study investigates the effects of climate warming on the taxonomic, network, and functional profiles of soil bacterial polyphosphate-accumulating organisms, leveraging fluorescence-activated cell sorting and single-cell Raman spectroscopy. Climate warming enhances both taxonomic and functional biodiversity of polyphosphate-accumulating organisms via biotic interactions and environmental filtering, with observed functionality-biodiversity relationships supporting the functional redundancy theory. Furthermore, polyphosphate-accumulating organism network complexity and stability rise under warming with strengthened positive relationships, supporting stress gradient hypothesis and the belief that complexity begets stability. Finally, polyphosphate-accumulating organisms are significantly correlated to key ecosystem functioning in carbon and phosphorus cycling under warming. Our study suggests that preserving polyphosphate-accumulating organism communities is crucial for maintaining soil ecosystem functioning and sustainable phosphorus management in a warming world and opens avenues for predicting the responses of other functional microbial groups to climate change, beneficially or maliciously.

## Introduction

Global climate change is reshaping soil microbial communities and profoundly impacting nutrient cycles, which are essential for maintaining ecosystem functioning and supporting diverse life forms [1–9]. Although extensive research has focused on carbon (C) and nitrogen cycling [8, 10, 11], the impacts of climate warming on soil phosphorus (P) cycling remain in its infancy. This gap is critical, as P is an essential element for all living organisms, agricultural productivity, and ecosystem functioning but non-renewable with limited global reserves [7, 12]. Current studies suggest that climate warming depletes soil P reserves through enhanced weathering [7, 13], but the role of microorganisms in regulating P availability has been largely overlooked. Only limited studies showed that long-term climate warming reduced total and bioavailable soil P pools via indirect bulk-level microbial activity [13, 14]. This underscores the critical yet often undervalued role of functional microorganisms in driving soil P dynamics under changing climates, calling for urgent exploration of how microbial biodiversity and soil P cycling respond to global climate change.

To better understand microbial regulation of soil P under climate change, it is essential to examine the key microbial groups involved in P cycling. Various phenotypic groups of P-cycling microorganisms are present in soil and play crucial roles regulating P dynamics [7, 12, 13, 15, 16]. Among these groups, polyphosphate-accumulating organisms (PAOs) have recently emerged as significant player in soil P cycling [17]. As the sole biological source of polyphosphate (polyP), PAOs are capable of accumulating luxury intracellular polyP and releasing bioavailable P, contributing up to 78% of total P pool in natural soil and up to 93% in polyP-fertilized soils [18–20]. Beyond serving as a P buffer and reserve, their versatile roles also extend to promoting sustainable agriculture and driving the coupled C-nitrogen-phosphorus cycling within soil ecosystems [17, 18, 21, 22]. Although phosphate-solubilizing bacteria and fungal PAOs have been recognized for enriching soil bioavailable P [14] or forming symbiotic relationships with plants [21, 22], the study of soil bacterial PAOs remains nascent largely due to challenges in detecting and identifying yet-uncharacterized PAO strains. Therefore, despite their recognized importance, the identity and ecological roles of soil bacterial PAOs, especially their response to climate warming, remain largely unexplored.

Although bulk techniques like metagenomic sequencing have provided insights into microbial diversity and genetic potential, [9] they can fail to capture the active metabolic activity of PAOs at the single-cell level. These traditional methods biased the functional contributions of individual PAO cells due to a major portion of microbial diversity (>40%) from extracellular DNA, dead cells, or dominant populations [23]. Novel biotechnologies like fluorescence-activated cell sorting (FACS) and single-cell Raman spectroscopy (SCRS) overcome these limitations by directly assessing the active metabolic activity of individual cells in their native habitats in a culture-independent manner [24, 25]. Both FACS and SCRS can simultaneously detect multiple biomarkers, such as intracellular polyhydroxyalkanoates (PHA) and polyP, enabling the study of active PAOs for their versatile adaptation to fluctuating environments. FACS allows for the exploration of taxonomy-level biodiversity and biotic interactions by sorting PAOs based on PHA and polyP content, [25] whereas SCRS provides a full-spectrum profile of PAO functionality, detecting molecular fingerprints that reveal their active single-cell metabolism [26, 27]. These advanced technologies offer a comprehensive view of PAO contributions to P cycling and uncover underlying adaptation strategies in response to climate change.

As climate warming increasingly imposes niche differentiations on microbial communities, several ecological theories provide frameworks for predicting its effects at a bulk community level. The environmental filtering theory, biotic interaction effects on taxonomic diversity, and the stress gradient hypothesis (SGH) collectively suggest that climate warming will drive microbial communities toward greater specialization and reduced biodiversity at the bulk community level, whereas enhancing positive biotic interactions [28–30]. Compared to these deterministic processes, the role of stochastic processes is relatively minor [31]. These predictions have been empirically verified in grassland ecosystems [1, 2]. Although these theories offer insights at the bulk community level, predicting changes within specific functional groups, like soil bacterial PAOs, remains challenging. Functional redundancy theory and biodiversity-ecosystem function theory predict that even with reduced taxonomic diversity, functional diversity and ecosystem processes like nutrient cycling may remain stable, though prolonged stress may eventually impair functional capacity [5, 6, 32, 33]. Hence, more empirical research is needed to understand the specific effects of climate warming on PAO biodiversity, function, and their influence on ecosystem resilience.

**Figure 1:**
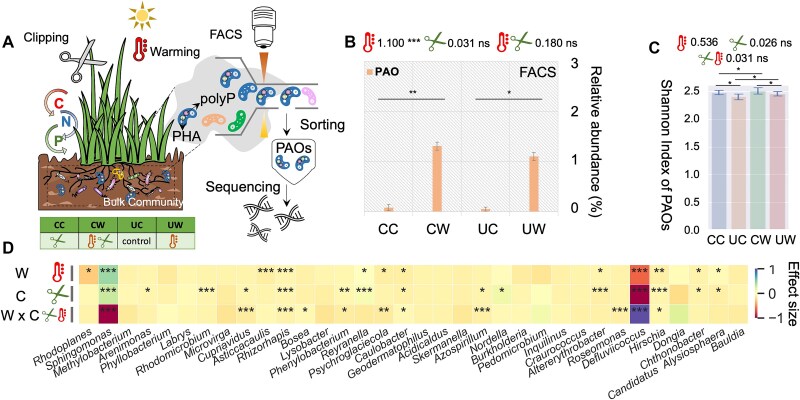
Impact of climate warming and annual clipping on the taxonomic diversity of soil bacterial phosphorus-accumulating organisms. (A) Function-targeted fluorescence-activated cell sorting (FACS) combined with amplicon sequencing to identify the PAOs that are actively accumulating both polyP and PHA. (B) Relative abundance of PAOs measured by FACS counting techniques. (C) Shannon biodiversity index for soil PAOs at taxonomic genus level in response to climate warming and annual clipping. (D) Renormalized effect size ($\beta \in \left[-1,1\right]$) of climate warming and annual clipping on individual PAO taxa. Note: **CC** denotes annual clipping with no climate warming, **UC** denotes control group with neither clipping nor warming, **CW** represents climate warming with annual clipping, and **UW** represents climate warming without annual clipping. The effect sizes of warming, clipping, and their interaction are indicated above the figure b and c, representing the magnitude of their influence on the respective measured variables. ^*^means P < .05, ^**^denotes P < .01, ^***^denotes P < .001, ns denotes not significant. All the following sessions use the same rule for significance label and the definitions of CC, CW, UC, UW.

This study aims to address these knowledge gaps by investigating how climate warming influences the taxonomic biodiversity, biotic interactions, and functional redundancy (FR) of soil active bacterial PAOs, as well as their contributions to ecosystem functioning. Grounded in ecological theories of environmental filtering, biotic interactions, and FR, we herein explore the scientific question: “How does climate warming affect the taxonomic and functional diversity of PAOs and their contribution to soil carbon and phosphorus cycling”. To test our hypothesis, a novel multi-tiered approach (Figs. 1a and 3a) is developed to study both the functionality and taxonomy of soil bacterial PAOs by integrating FACS, SCRS, amplicon sequencing, and computational analytics. Our findings show that: (i) Climate warming enhances PAO biodiversity both taxonomically and functionally through biotic interactions and environmental filtering; (ii) Functional redundancy is maintained in the PAO community under warming; and (iii) Warming strengthens the positive correlation between PAOs and ecosystem functioning. These results collectively demonstrate that PAOs contribute positively to grassland ecosystems under climate warming, even as the overall biodiversity of the bulk community declines. By uncovering these mechanisms, this research seeks to provide insights into how future climate shifts may affect soil nutrient pools and ecosystem stability, offering valuable predictions for sustainable P management in a warming world.

## Results

### Effects of climate warming on the taxonomic biodiversity of PAOs

Field experiments were designed to primarily investigate the effects of climate warming on soil microbial communities, with annual clipping as a secondary factor. The treatments were divided into four groups: CC (clipped, control), UC (unclipped, control), CW (clipped, warming), and UW (unclipped, warming) (Figs. 1a & SI Fig. S1 & Supplementary Note A). To assess the effects of climate warming on soil bacterial PAOs, we used FACS to sort PAOs that accumulated polyP and polyhydroxyalkanoates (PHA), followed by 16S rRNA gene amplicon sequencing and bioinformatics analysis to identify PAOs and evaluate changes in their taxonomic profiles (Figs. 1a & SI Fig. S2a, b). The FACS enrichment efficiency was high across most PAO taxa (SI Fig. S2c, d), allowing for a detailed analysis of their diversity and community composition. This pipeline offered an in-depth exploration of taxonomic identities and the shifts in diversity and composition in response to climate change. To quantify the effects of climate warming on PAO community with or without annual clipping, linear mixed-effects models (LMMs) were performed as previously described [1]. The effect size ($\beta$) of LMMs represented the directions and magnitudes of the treatment effects.

The LMMs showed that warming significantly impacted the soil microclimate by increasing temperature and reducing moisture, affected geochemical properties (e.g. decreasing soil pH and total phosphorus [TP]), and significantly increased plant biomass (SI Fig. S3). In terms of the bulk soil microbial community, our results showed a reduced taxonomic biodiversity by 5% (*P* < .001). Regarding the effects on PAOs, climate warming increased the relative abundance of PAOs by 1.5% significantly ($\beta =1.100,$  *P* < .001) and enhanced taxonomic biodiversity ($\beta =0.536,P<.05$) (Fig. 1b, c). Moreover, climate warming positively affected a substantial portion ($33.3\%,(\mid \beta \mid >0.1$) of the PAO populations (Fig. 1d). For instance, *Sphingomonas*, known for improving P availability in soil and accessing to aromatic soil organic C stocks [34], was significantly enhanced (*P* < .001). As for annual clipping and the interaction of warming and clipping, they did not significantly affect the relative abundance and the Shannon diversity of PAOs significantly (Fig. 1b, c & Supplementary Note B). Altogether, these results suggested that climate warming is the major factor to enhance the taxonomic biodiversity of the PAO community, strongly filtering certain species like *Sphingomonas*.

### Impacts of climate warming on microbial networks of PAOs

Understanding the effects of climate warming on biotic interactions (e.g. cooperation, competition, resource sharing) is crucial for determining how PAO communities maintain functionality and ecosystem services beyond mere biodiversity changes. To evaluate these effects, we employed a taxa correlation-based non-parametric approach for PAO network construction (details in Supplementary Note C & Table S1-S2). [2, 35–38] Moreover, PAO networks exhibited significant differences from the broader microbial community networks, and PAO networks varied significantly across different treatments, as demonstrated by Adonis, MRPP, and ANOSIM tests (SI Fig. S4). These findings indicate that PAOs respond to climate change in a more distinct and pronounced manner compared to the overall bacterial network.

**Figure 2:**
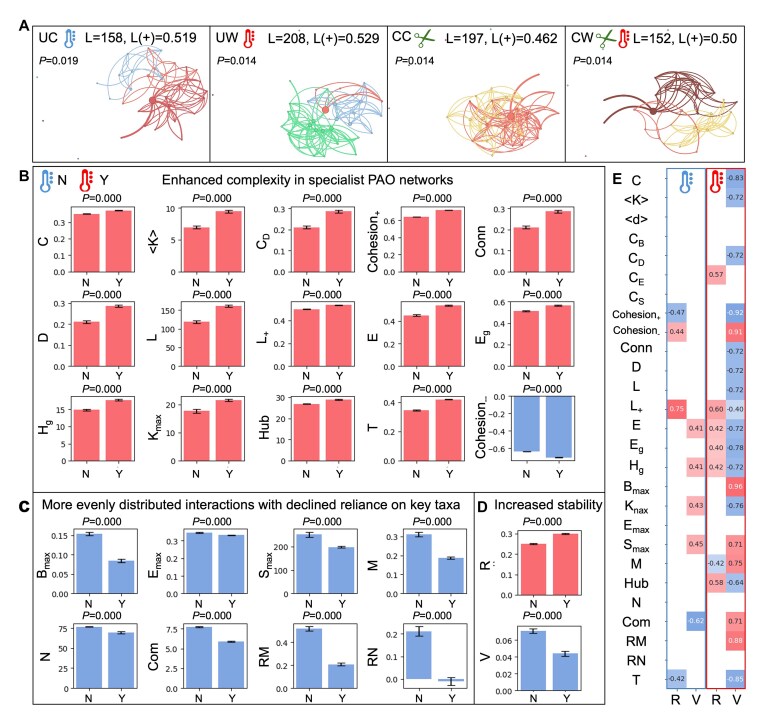
Impact of climate warming and annual clipping on network structure of soil bacterial PAOs. (A) Visualization of representative PAO co-occurrence networks under different treatments. Each network represents the interactions between PAO taxa, where nodes are taxa and edges indicate significant correlations between them. The number of edges ($L$) and positive edge ratios (${L}^{+}$) are indicated for each network, with the associated p values showing the statistical significance of treatment effects on network structure. (B) Climate warming significantly enhances network complexity in both warming (Y) and ambient temperature (N) treatment. Key metrics (n = 12) such as clustering coefficient ($C$), average degree ($<k>$), centralization of degree (${C}_D$), positive cohesion ($Cohesio{n}_{+}$), density ($D$), number of edges ($L$), positive edge ratio (${L}_{+}$), efficiency ($E$), and geodesic efficiency (${E}_g$), harmonic geodesic distance (${H}_g$), maximal degree (${K}_{max}$), number of hubs ($Hub$), and transitivity ($T$) all increase, indicating stronger interactions, higher connectivity, and improved efficiency within the PAO network. These changes suggest that the PAO network becomes more interconnected and capable of maintaining positive interactions under warming conditions. (C) Declined role heterogeneity in the PAO network under warming conditions, as shown by decreases in betweenness centrality (${B}_{max}$), eigenvector centrality (${E}_{max}$), stress centrality (${S}_{max}$), modularity ($M$), nestedness ($N$), connectance ($Conn$). The decline in relative modularity ($RM$) indicates a breakdown in distinct sub-communities, although the negative relative nestedness ($RN$) suggests a loss of hierarchical interaction structure, implying reduced specialization in network interactions. Together, these suggest that the PAO network under warming conditions is becoming more evenly distributed, meaning that no single group of taxa or type of interaction dominates, which confers stronger network stability under random taxon extinction. (D) Climate warming increases the overall stability of the PAO network. Robustness ($R$) increases, although vulnerability ($V$) decreases, showing the enhanced resilience of the network to disturbances under warming conditions. (E) Pearson correlations (*r* ≥ 0.6, *P* < .05) between complexity and stability metrics, with warming (red box) and ambient temperature (blue box) conditions. Under warming, 27 significant correlations were observed compared to only nine significant correlations under ambient conditions, highlighting the critical role of complexity in enhancing network stability under warming. More details in *supplementary note C.*

Based on SGH theory, we hypothesized that increasing environmental stress (e.g. climate warming), would enhance collaborative interactions among PAOs, leading to more interconnected and cooperative network structures. Indeed, under warming, PAO networks displayed significantly enhanced complexity across multiple structural metrics (Supplementary Note C & Table SC2), suggesting a more interconnected and cohesive community organization. Warming increased clustering coefficient ($C$), average degree ($<k>$), network density ($D$), network efficiency ($E$) and geodesic efficiency (${E}_g$), alongside a higher positive edge ratio (${L}_{+}$) and positive cohesion ($Cohesio{n}_{+}$), indicating stronger interactions dynamics and higher connectivity among PAO taxa (Fig. 2a, b). Specifically, the rise in ${L}_{+}$ and $Cohesio{n}_{+}$ suggests that mutualistic and cooperative interactions between PAOs are enhanced under warming, leading to a more collaborative network structure. Moreover, certain PAO genera (e.g. *Cupriavidus*, *Azospirillum*), known for their abilities to solubilize inorganic phosphates and degrade organic compounds [39], were strongly correlated with key network metrics (SI Fig. S5), highlighting their significant roles in shaping network complexity and stability under warming. Altogether, these results imply that warming not only fosters a more complex network structure but also facilitates more efficient and cooperative interactions among PAO members.

Despite this rise in complexity, role heterogeneity within the PAO network significantly declined under warming, showing a less reliance on a specific single taxon and confers stronger network stability such as under random taxon extinction (Fig. 2c). For instance, betweenness centrality (${B}_{max}$), eigenvector centrality (${E}_{max}$), and modularity ($M$) all decreased, indicating a less distinct sub-communities and a more homogeneous interaction landscape. The loss of hierarchical interaction structure, reflected by negative relative nestedness ($RN$), suggests that no dominant taxa or groups. This shift toward a more evenly distributed network coincided with an increase in robustness ($R$) and a reduction in vulnerability ($V$), highlighting greater resilience to climate change-induced perturbations (Fig. 2d). Furthermore, the strengthened correlation between complexity and stability metrics under warming (Fig. 2e) supports the macroecological theory that “complexity begets stability.” Together, these results suggest that climate warming positively fosters more complex, cohesive, and stable PAO networks with enhanced cooperative interactions and resilience, likely due to their adaptative advantages such as additional energy and C reserves under environmental stressors.

### SCRS-enabled functional characterization of PAOs and their linkage to taxonomies

**Figure 3:**
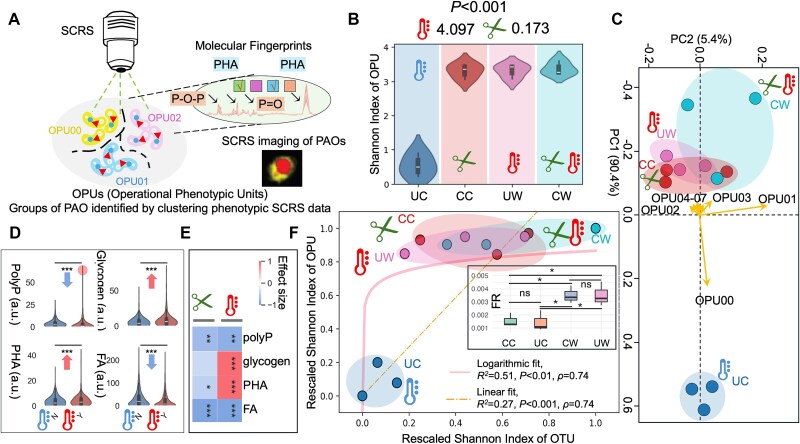
Single-cell functional analysis of PAOs in response to climate warming and annual clipping. (A) Function-targeted SCRS reveals intracellular molecular fingerprints of PAOs, allowing the inference of OPUs for quantification of functional diversity. One OPU represents one functional group of single cells clustered based on their SCRS-measured molecular characteristics, a similar concept to the OTUs in taxonomic analysis. b, the abundance of functional OPUs in PAOs in response to climate warming and annual clipping with effect size computed by LMMs. c, PCA biplots of OPUs show the separation of PAOs based on OPUs, indicating how climate warming and annual clipping influence different OPUs. d, the distribution of key intracellular metabolites (polyP, glycogen, PHA, and FA) across warming (Y) and ambient temperature (N). E, effect sizes of the treatment impacts (warming and clipping) on intracellular metabolite levels. f, the correlations of Shannon index of taxonomic OTUs and Shannon index of functional OPUs for the PAOs with linear and logarithmic fitting curve. To further validate the existence of functional redundancy (FR), FR is computed across treatments using Tax4Fun2 [45] and the plot is embedded. To optimize the sorting workflow and ensure sufficient sample quantities for FACS, six soil replicates per treatment were randomly pooled into three pooled replicates, which has been validated to introduce no statistical bias [76]. *Note*: *A total of 4 800 single cells were retrieved for SCRS analysis on four treatments, each treatment consists of six soil replicates and 2 SCRS-level drop replicates, and 100 phenotypically confirmed PAO single cells were measured for each drop. The single-cell sampling sizes have been verified to be sufficient (**Supplementary Note D*). ^*^means P < .05, ^**^denotes P < .01, ^***^denotes P < .001. All the following sessions use the same rule for significance label and the definitions of CC, CW, UC, UW.

To better understand the role of PAOs in ecosystem functioning and biogeochemical processes, the functional diversity of the PAO community was further studied, as climate warming-induced genotypic dynamics (e.g. increased taxonomic diversity and network stability) cannot fully capture critical functional shifts. Here, SCRS was employed to provide culture-independent and single-cell phenotypic molecular fingerprints, enabling direct quantification of PAO community functional diversity through the identification of operational phenotypic units (OPUs) (Fig. 3a and Supplementary Note D) [24, 26, 27]. An OPU represents a functional group of single cells clustered by SCRS-measured molecular traits, analogous to operational taxonomic units (OTUs) in taxonomic analysis but focused on functional characteristics. The functional diversity of PAOs was then calculated as the Shannon diversity of OPUs, consistent methodology with taxonomic biodiversity.

Seven OPUs emerge as dominant and 80% of OPUs are preserved within all the PAO community amongst different treatments (SI Fig. S6a, b & Supplementary Note D). This field has been subjected to continuous warming and clipping treatments for over 20 years since 1999, allowing long-term ecosystem shifts to shape microbial community structure and function (SI Fig. S1 & Supplementary Note A). As a result, the negative control US is expected to diverge significantly from the treated fields (UW, CC, CW). Consistently, under climate warming and clipping treatments, the functional diversity of PAOs significantly increased, with LMMs showing that warming was the primary driver (β = 4.097, *P* < .001), whereas clipping had negligible effects (β = 0.173, *P* > .05) (Fig. 3b). To investigate the role of each OPU under climate change and their associations with C- and phosphorus-related molecules, our results suggested that OPU00-OPU02 were predominantly associated with fatty acids (FA) and contributed to the clipping and control groups, whereas OPU03-OPU07 were linked with glycogen and were more prominent under warming conditions (Fig. 3c & SI Fig. S6c). Furthermore, warming increased glycogen (β = 3.632, *P* < .001) and PHA (β = 1.175, *P* < .001) accumulations, whereas polyP (β = −0.875, *P* < .005) and FA (β = −10.895, *P* < .001) decreased, though the elevated highest polyP values suggest that some PAOs adapt by accumulating more polyP despite the overall decline (Fig. 3d, e). Qualitatively, polyP, a key phosphorus and energy storage molecule, is consumed under increased temperatures to possibly provide both energy and phosphate for enhanced metabolic activity. In wastewater ecosystems, polyP and FA consumption are typically associated with enhanced PHA accumulation, conferring a competitive advantage to PAOs under fluctuating environmental conditions [17, 40]. Specifically, FA serves as a C source for PHA synthesis, while a portion of the assimilated C is redirected toward glycogen replenishment [41, 42]. Therefore, the observed decrease in FA and the increased accumulation of PHA and glycogen likely reflect microbial metabolic responses to climatic environmental filtering, although quantitative interpretations of the changed magnitudes in different biopolymers would require further detailed molecular verifications with pure cultures. Altogether, these molecular changes suggest that under climate warming, PAOs shift towards accumulating more C storage compounds (e.g. glycogen and PHA), whereas selectively using polyP and FA as energy and C sources, providing them with better competitive advantages to survive and function more efficiently in nutrient cycling under climate stress.

To better understand the relationships between functional diversity and taxonomic structure of PAO community, a canonical correspondence analysis-based variance partitioning analysis (CCA-based VPA) was performed to show that 80% of the OPU structures are explained by taxonomic biodiversity and biotic interactions (*P* < .05, SI Fig. S7). Moreover, certain OPUs have strong correlations with biotic interactions, such as OPU02 (62% of network metrics showed significant correlations) and OPU03–07 (13.8%–41.4%), indicating the critical role of interactions in shaping functional diversity (SI Fig. S8). These findings align with niche theory and community assembly theory, where functional diversity is shaped by environmental filtering and biotic interactions acting on taxonomic compositions [30, 31, 43]. Finally, based on the climate-induced increase in both taxonomic and functional Shannon index values within the PAO community, a logarithmic fit (*R^2^* = 0.51, *P* < .01) outperforms a linear fit (*R^2^* = 0.27, *P* < .001) in describing the relationship between functional and taxonomic diversity (Fig. 3f). This suggests the presence of FR within the PAO community, which confers greater resilience by allowing it to maintain functionality even with the random loss of certain PAO taxa. We further quantified the existence of FR and it showed that climate warming significantly increased FR compared to UC and CC (*P* < .05) using R package Tax4Fun2 (Fig. 3f) [32, 33, 44, 45]. Furthermore, this heightened diversity in soil PAOs may enhance ecosystem resilience and functionality, as PAOs play a significant role in both C and phosphorus cycling, a principle supported by the diversity-stability theory, which posits that diverse ecosystems are more stable and provide a broader range of ecosystem services [28, 33, 44].

Altogether, these findings suggest that climate warming increased functional diversity and retained FR within the PAO community, with shifts towards higher glycogen and PHA accumulation and reduced polyP and FA levels, providing resilience to ensure the maintenance of critical C and phosphorus cycling functions in ecosystems.

### Mechanisms underlying increased PAO biodiversity and their linkages to ecosystem functioning

**Figure 4:**
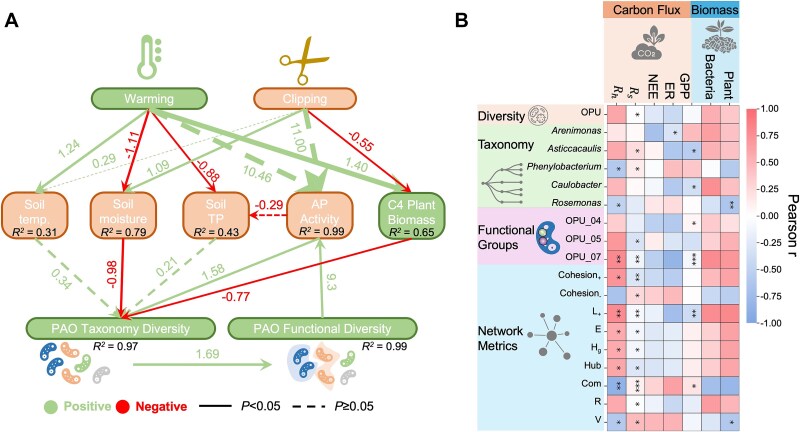
Environmental drivers of PAO biodiversity under climate warming and its linkage to ecosystem functioning. (A) SEM path diagram illustrating the direct and indirect effects of climate warming and annual clipping on soil microclimate, total phosphorus (TP), and their subsequent impacts on C4 plant biomass, alkaline phosphatase (AP) activity, and PAO diversity. The relationships were depicted using green and red arrows, indicating positive and negative relationships, respectively. Solid or dashed lines represented significant (*P* < .05) or non-significant relationships. The standard path coefficients were denoted by numbers near the pathway arrow. Line width corresponds to the absolute value of the standard path coefficients. The explained variance (*R^2^*) for each endogenous variable is indicated within the respective nodes. The model is well fitted with results of RMSEA (root mean square error of approximation) = 0.05, CFI (comparative fit index) = 0.993, χ^2^ = 28.76, d.f. = 28, *P* = .425 (it shows a good fitness, i.e. hypothetical SEM model is equal to established SEM model). (B) Pearson correlation coefficients ($r$) between key ecosystem functioning (i.e. NEE, GPP, ER, soil respiration (R_s_), soil heterotrophic respiration (R_h_)) and various PAO-related metrics (i.e. diversity, PAO taxon, OPU groups, network metrics). Only rows with at least one significant correlation are shown, with full details available in Supplementary Note E.

As we demonstrated before, warming-induced taxonomic biodiversity increase could be due to enhanced cooperative biotic interactions and abiotic environmental filtering, which further confers the FR within PAO community. Therefore, under warmer conditions, it is significant to investigate how shifts in environmental variables, such as soil temperature, moisture, and phosphorus availability, directly affect the taxonomic and functional diversity of PAOs and their broader impact on ecosystem functioning. The structural equation modeling (SEM) analysis illustrates the direct and indirect effects of climate warming and annual clipping on key environmental and biodiversity variables (Fig. 4a). Warming significantly increased soil temperature (standardized path coefficient, $b=1.24$) and decreased soil moisture ($b=-1.11$), leading to reductions in total phosphorus (TP) ($b=-0.88$) and increase in alkaline phosphatase (AP) activity ($b=10.46$). These changes in soil geochemistry and moisture levels, in turn, influenced PAO taxonomic diversity (${R}^2=0.97$) and PAO functional diversity (${R}^2=0.89$). Additionally, AP activity showed a strong negative impact on TP ($b=-0.29$), which suggests that as AP activity increases via the chain of increased PAO taxonomic biodiversity ($b=1.69$) and functional diversity ($b=9.3$), more organic phosphorus is broken down and converted into bioavailable forms for higher intracellular polyP accumulation (Fig. 3d, e), leading to a reduction in TP as it is utilized by the ecosystem. Moreover, the chain of interactions shows that clipping ultimately contributed to an increase in PAO functional diversity by reducing C4 biomass ($b=-0.55$), which in turn increased taxonomic biodiversity ($b=-0.77$), leading to a compensatory rise in PAO functional biodiversity ($b=1.69$). Overall, these results confirm that taxonomic diversity is shaped by biotic interactions and environmental filtering, particularly via soil moisture and TP, and that functional diversity, driven by taxonomic biodiversity, plays a key role in ecosystem functioning.

Another key consideration is how the warming-induced changes in PAO community influence broader ecosystem functioning. Consistent with the enhanced diversity and networks of PAOs (Figs. 1–3), warming also improved ecosystem functioning, reflected by the positive correlations between PAOs and gross primary production (GPP, Pearson $r$) and the negative correlations between PAOs and net ecosystem exchange (NEE) and ecosystem respiration (ER) (Fig. 4b & SI Fig. S10 & Supplementary Note E). Moreover, PAOs play vital roles in both soil C and phosphorus cycling due to their ability to accumulate more intracellular C polymers (i.e. glycogen and PHA) via depletion of polyP, contributing to increased soil organic C (Fig. 3d, e & Supplementary Note E). These storage compounds act as buffers, allowing PAOs to regulate nutrient availability under fluctuating conditions, supporting both soil P dynamics and C storage. Altogether, these suggest that PAOs positively contribute to the ecosystem C sink capacity and P cycling by enhancing C sequestration and acting as a P buffer pool.

## Discussion

### Warming increases diversity and ecological stability of PAO communities in grassland ecosystems

Climate warming induces divergent microbial responses, with overall biodiversity declining while PAO diversity increases (Fig. 1). This pattern aligns with the SGH and environmental filtering theory, which suggest that intensified environmental stress selects for specialized taxa capable of thriving under altered conditions [28–30]. In this study, PAOs, with their coupled P and C cycling capabilities, gain a competitive advantage under long-term warming, displacing generalist taxa in the bulk microbial community. Network analyses further reveal that warming increases PAO interaction complexity, leading to higher clustering coefficients, average degrees, and network densities (Fig. 2). Similar enhanced network complexity and stability has been observed in the bulk microbial community of grassland ecosystems under long-term climate warming [2]. These structural changes enhance P and C cycling efficiency, reinforcing microbial stability under environmental fluctuations. By fostering a highly interconnected microbial network, warming stabilizes P turnover, buffering against climate-induced nutrient imbalances. Previous studies demonstrated similar observations in C-cycling and nitrogen-cycling microbial community, such as increased genes involved in degrading recalcitrant compounds, and enhanced labile-C degradation, N fixation, and denitrification rates under warming [5, 9, 46]. Moreover, FR was preserved within PAO communities to ensure persistence of P cycling processes despite taxonomic structure shifts under long-term warming (Fig. 3). The redundant functional groups act as ecological buffers, maintaining ecosystem function even when specific taxa decline. By sustaining nutrient cycling despite climate-induced disturbances, FR stabilizes ecosystem services and enhances long-term resilience. Similar observations have been reported that climate warming promotes taxonomic redundancy and positively correlates with FR across marine and soil ecosystems [47, 48]. Altogether, PAO communities hold significant roles in grassland ecosystem resilience, mitigating the impacts of environmental stressors and reinforcing the need for ecosystem management strategies that preserve microbial functional diversity.

### SCRS-FACS phenotyping platform is transferable to other microbial systems under climate change

The SCRS-FACS platform uniquely enables direct phenotyping of metabolically active PAOs at the single-cell level (Figs. 1 and 3), an essential capability that sequencing alone cannot provide. While metagenomics and amplicon sequencing have been utilized to reveal the taxonomic diversity of PAOs, they fail to distinguish active PAOs from inactive or previously unknown PAO taxa and cannot resolve functional heterogeneity within closely related populations [40]. In contrast, SCRS-FACS sorts cells based on their metabolic state, providing direct functional insights rather than inferred genomic potential. This real-time metabolic profiling is crucial for understanding microbial functional dynamics under climate change, revealing ecological roles that remain hidden in bulk sequencing analyses. Integrating SCRS-FACS with other omics approaches will allow future studies to build a mechanistic understanding of microbial community function in response to climate change. Beyond PAO identification, the SCRS-FACS platform holds broad potential for studying microbial functional dynamics across diverse ecosystems and environmental perturbations. This methodology can be applied to other key microbial groups, such as nitrogen fixers, methane oxidizers, and sulfate reducers, across diverse ecosystems. The high-resolution insights from SCRS-FACS enable real-time tracking of microbial responses to stressors like warming, pollution, and land-use change, supporting future research in microbial traits, multi-omics integration, and ecosystem modeling. By extending SCRS-FACS applications across ecosystems, we can gain a deeper understanding of functional microbial contributions to global biogeochemical cycles and improve strategies for ecosystem management under climate change.

### Global climate change factors drive soil nutrient cycling

Beyond warming, multiple climate change factors, such as altered precipitation patterns, elevated atmospheric carbon dioxide (CO_2_), atmospheric nitrogen deposition, profoundly impact soil nutrient cycling [5]. For instance, changes in precipitation regimes influence microbial access to P and C, as soil moisture fluctuations regulate enzymatic activity, nutrient diffusion, and microbial community structure. Drought conditions can significantly alter microbial structures and P transformations [49] and elevated CO_2_ levels can modulate nutrient cycling by stimulating plant root exudation, increasing organic C inputs, and reshaping microbial interactions [5, 50]. Long-term climate change effects also alter the balance between microbial immobilization and mineralization of P, impacting bioavailability and plant uptake [12–14]. In response to environmental variability, microbial communities exhibit adaptive functional traits, such as enhanced P storage through polyP accumulation [17]. These factors may collectively influence PAO dynamics, potentially reinforcing or counteracting the effects of warming on microbial P cycling. Understanding how multiple climate drivers interact to shape PAO functional diversity is crucial for predicting soil fertility trajectories and informing sustainable land management strategies. Future research should integrate multi-factorial climate manipulations with advanced phenotyping-genotyping approaches, such as SCRS-FACS platform, to unravel microbial functional responses to complex environmental stressors.

## Materials and methods

### Experimental site and design

The soil samples were collected from a tall-grass prairie ecosystem in the US Great Plains in Central Oklahoma (34°59’ N, 97°31’ W), a long-term experimental field that has been treated and monitored since 1999, as described in previous studies [9, 51]. Briefly, the experimental sites were divided into two ecosystems, an unclipped tall-grass prairie ecosystem and a clipped tall-grass prairie ecosystem (SI Fig. S1 & Supplementary Note A). The clipping was to mimic hay harvesting, which was commonly practiced in the area. In order to study the impacts of climate warming, the primary treatment factor is the warming. Each ecosystem was sub-divided into two plots, a control plot—no artificial heating, and a warmed plot. The warming plots have been continuously warmed at 2°C above natural temperature since November 1999.

A total of 24 soil samples (ca. 10 g soil per sub-division, six replicates for each treatment, total of four treatments) were packed in a 4 oz clear Whirl-Pak bags (Nasco, WI, USA) and shipped in an icebox. Upon the arrival, 1 g of samples were transferred to 2 ml micro-centrifuge tubes and stored at −80°C for DNA analysis. The remainder of the samples were stored at 4°C for subsequent chemical and biological analysis.

### Field measurements and soil chemical analysis

Ecosystem respiration (ER), NEE, soil total respiration (R_s_), and soil heterotrophic respiration (R_h_) were measured once or twice a month between 10:00 and 15:00 (local time) as described previously [1, 2]. The ecosystem GPP was then estimated by subtracting NEE from ER. The above-ground plant biomass (C3 and C4 species) was measured by a modified pin-touch method as described previously [52].

#### Water content, loss on ignition, organic matter, and pH measurement

The soil water content was measured by drying the soil at 105°C for 24 h. The dried soil was furnaced for 4 h at 550°C for loss on ignition (LOI). The % LOI is converted to % soil organic matter using Eqn. 1 [53].


(1) \begin{equation*} \% OM=\left(\% LOI\times 0.7\right)-0.23 \end{equation*}

The pH was measured by placing pH meter in a 1:2 soil: deionized water suspension [53].

#### Soil phosphorus measurement

The P contents were measured using Stannous Chloride Method with UVmini-1240 Spectrophotometer (Shimadzu, Japan). Soil P was extracted by digesting furnaced soil samples in HNO_3_:HClO_4_ at a 1:10 ratio for 10 min at 160°C measured by Cornell Nutrient Analysis Laboratory.

#### Soil nitrogen measurement

Soil NO_3_^−^ and NH_4_^+^ was measured with AS22 mounted Dionex ICS-2400 (Thermo Scientific, MA, USA), and CS16 mounted Dionex ICS-2400 (Thermo Scientific, MA, USA), respectively. NO_3_^−^ and NH_4_^+^ were extracted by suspending fresh soil samples in 0.01 M CaCl_2_ at a 1:10 ratio and shaking them for 1 h at 200 rpm under room temperature. Extractants were centrifuged for 10 min at 1, 200 g, and the supernatants were filtered with a 0.2 μm filter for IC measurement. Here, total inorganic nitrogen (TIN) consists of NO_2_^−^, NO_3_^−^, and NH_4_^+^.

#### Alkaline phosphatase activity test

To measure the alkaline phosphatase (AP) activity, fresh soil (1 g) sample was mixed with 150 ml of 50 mM sodium bicarbonate buffer and blended for 30 minutes. The resulting slurry was transferred to a stir plate and gently mixed. Subsequently, 200 μl of the slurry was transferred to a 96-well plate. To each well, 50 μl of 200 μM 4-Methylumbelliferyl phosphate (4-MUB-phosphate, Sigma–Aldrich #M8883) was added as the substrate. The 96-well plates were then incubated at 25°C for 2 hours, and fluorescence measurements were conducted using a Synergy H1 Microplate Reader (Biotek, VT, USA) at 365 nm excitation and 450 nm emission wavelengths.

#### Phospholipid fatty acid

Lipids were extracted by the modified Bligh-Dyer method in the soil samples as previously described [1, 54]. Briefly, each 2 g freeze-dried soil sample was treated with a methanol, chloroform, and K_2_HPO_4_ buffer mix, and chloroform layers were extracted for phospholipid separation using silicic acid chromatography. These were then converted to fatty-acid methyl esters and analyzed via gas chromatography (Agilent 6890 N) using a MIDI Sherlock system and Agilent Chemstation software The total bacterial biomass of the soils was calculated as the total phospholipid fatty acid of all bacterial groups, i.e. the sum of the biomass of Gram-negative bacteria, Gram-positive bacteria, *Actinobacteria* and anaerobic bacteria.

### PAOs identification and taxonomic analysis

#### Definitions of PAOs in this study

In this study, PAOs are characterized as microorganisms that accumulate both polyP and PHAs for the following reasons. Model PAOs, such as *Candidatus Accumulibacter, Pseudomonas, Dechloromonas* [40, 55, 56], exhibit metabolic coupling between PHA and polyP pathways. Although some PAO taxa (e.g. *Tetrasphaera*) may not exhibit such coupling, the *Candidatus Accumulibacter* metabolic model is still widely accepted in EBPR ecosystem. Moreover, in natural ecosystems such as soil, the phylogenetic identities and diversity, ecological roles, and functional significance of PAOs are largely unknown, thus the definition of PAOs in natural ecosystems is yet to be developed. Previous studies indicated that PHA is recognized as one of the dominant C storage biopolymers, with multiple roles in C nutrient cycling and dependence with other nutrient cycling [57, 58]. For example, the concentrations of PHA (~1–4 μg-C/g soil) are positively correlated to the availability of soil C and suppressed by N availability, leading to its suggested function as an alternative C storage mechanism to support stoichiometric balance [57, 59]. Lastly, our SCRS data of PAO cells in crop soil (unpublished) suggests that PHA co-accumulation was observed in up to 73.7% of polyP-positive cells and the Pearson correlation coefficient between polyP-PHA-accumulating cells and polyP-accumulating cells is 0.81 (*P* < .001), implying the likelihood and underlying biological metabolic linkage of the two biopolymers in soil systems. For instance, the simultaneous accumulation of polyP and C biopolymers improves microbial resilience to changing nutrient conditions, distinguishing specialized PAOs with surplus storage driven by resource availability from those accumulating polyP for stress response or cation sequestration [58, 60–62]. Therefore, we evaluate this specific sub-group of PAOs in this study, and further study is needed to expand from this specific sub-group of PAOs to varying PAO groups with accumulations of other biopolymers, but they are beyond the scope of this study.

#### Identification of bacterial PAOs via fluorescence-activated cell sorting and downstream 16S rRNA gene amplicon sequencing *microbial community* analysis

To quantify and sort soil PAOs, FACS instrumental settings and gating strategy were employed as previously published [63] and the detailed methodology was provided within Supplementary Note F. An overview of the method was provided as follows: soil samples were pretreated by cell detachment, separation, followed by cell fixation and triple-staining procedure targeting DNA, polyP, and PHA, using SYBR Green I, tetracycline hydrochloride, and Nile red, respectively. Following staining, FACS was performed to quantify and sort microbial subpopulations simultaneously positive for all triple biomarkers, which are indicative of PAOs defined in this study. The FACS protocol employed a combination of scatter- and fluorescence-based gating strategies to select single cells and exclude non-cellular particles as described in Supplementary Note F. Sorted cells were then subjected to genomic DNA extraction and 16S rRNA gene amplicon sequencing, enabling taxonomic profiling of bacterial PAOs at high resolution.

#### Single cell Raman spectroscopy for phenotypic characterization of bacteria PAOs

For single cell Raman spectroscopy (SCRS) analysis of PAOs [27], two replicates per treatment were prepared, with 100 single cells measured per replicate, which was deemed statistically sufficient based on kernel divergence algorithm as previously described [64] (Supplementary Note D and SI Fig. S12-S13). To align SCRS results with that of FACS, SCRS specifically detected bacterial PAOs by identifying peptidoglycan (726 cm^−1^, 1421 cm^−1^, 1573–1582 cm^−1^) [65], amino acid and protein components Amide I-III (1002 cm^−1^, 1220 cm^−1^, and 1657 cm^−1^) [66, 67], and PHA (840 cm^−1^and 1725 cm^−1^) [26, 27]. As for polyP, our previous studies have evaluated various factors that may impact the PolyP detection by SCRS, including polyP length and different counter metal ion [66, 68]. Li et al. showed that typical polyP has various mixture of counter ions, and they do not impact the polyP detection via SCRS. Based on our previous results, for polyP length greater than 6 (as the case for most polyP observed in environmental samples), the two signature peaks of polyP are wavenumber ranges 690–700 cm^−1^ (P-O-P) and 1168–1180 cm^−1^ (PO_2_^−^) for polyP [66, 68]. Specifically, microorganisms were separated from soil by shaking soil samples in a detergent mix (0.5% v/v Tween 20, 3 mM Na_4_P_2_O_7_, and 0.35% wt/v polyvinylpyrrolidone) for 30 min at 200 rpm, followed by Nycodenz solution and centrifuged (1.5 hour at 14 000 g, 4°C). Supernatants were washed four times with 1X PBS solution, each involving resuspension and centrifugation for 1 min at 14 000 g. Final solution (2 μl) was dried on a CaF_2_ slide (Crystran Ltd., Dorset, UK) as described previously [26, 27]. Single-cell Raman spectra were acquired using a Horiba Labram Evolution (Horiba, Kyoto, Japan) equipped with 50X objective (Olympus LMPLFLN 50X, Tokyo, Japan). Excitation was provided by a 532 nm diode laser. The spectra were collected from 400 to 2000 cm^−1^ with ND of 25%, acquisition time of 20 s, and 3 accumulation times.

### Statistical and network analysis for PAOs community

All data analytics were conducted using customized Python 3.10 and Linux scripts.

#### FACS-sorted PAOs enrichment community analysis

To quantify the enrichment of FACS-sorted PAOs, OTUs from amplicon analyses were preprocessed using Mothur [69]. Subsequently, OTUs were filtered based on specific criteria: those with a relative abundance below 0.01%, e-scores under 99, or lacking genus-level taxonomy were removed. Functional validation was then performed using PICRUSt2 [70], ensuring that selected OTUs contained genes related to both polyP and PHA synthesis and degradation (*ppk1, ppx, phaZ,* and *phaC*). This step aligned 16S rRNA-inferred genomic capabilities with the phenotypic accumulation of polyP and PHA, improving the reliability of the selected OTUs to represent PAO communities in the subsequent analysis.

#### Operational phenotypic unit clustering of SCRS-identified PAOs

Multivariate analysis of Operational Phenotypic Unit (OPU) clustering was carried out using the Raman spectra of all bacterial PAO single cells identified from the SCRS spectra. The OPU, analogous to the widely accepted OTU, is defined as functional subgroups derived from cluster analysis of SCRS spectra. Although OTUs are defined by nucleotide sequence similarities, OPUs are determined by the molecular fingerprint similarities revealed through SCRS data. Raman spectra were binned in 6 cm^−1^ intervals, and hierarchical clustering was conducted using cosine-based correlation distance and average linkage, with a cutoff value of 0.6 suitable for the high heterogeneity nature of soil microbial communities [71].

#### Diversity analysis

Shannon index $H$ was used to measure the diversity of FACS-sorted PAOs soil communities in the study, computed by Eqn. 2.


(2) \begin{equation*} H=-\sum_{i=1}^n{P}_i\ \log \left({P}_i\right), \end{equation*}

Where ${P}_i$ is the relative abundance of $i$-th taxon (or OPUs) in the sample with total of $n$ taxa (or OPUs).

#### FR analysis

The $\mathrm{FR}$ of PAO community across treatments is calculated using R package Tax4Fun2 [45]. Briefly, FR is quantified using a ratio of the sum of functional contributions across all taxa to the number of unique taxa contributing to the function, using Eqn. 3.


(3) \begin{equation*} FR=\frac{\sum_{i=1}^N{f}_i}{S}, \end{equation*}

Where ${f}_i$ denotes functional contribution of taxon $i$ to KEGG KO pathway [72], $N$ denotes the total number of taxa contributing to the function, $S$ represents the number of unique taxa in the community.

#### Treatment effects by LMMs analysis

LMMs were employed in this study to quantify the impacts of climate warming and clipping on PAO Shannon diversity and the diversity of individual taxonomic groups within the PAO communities. LMMs were chosen due to their ability to handle nested or repeated measures data, accounting for individual variation and differences between experimental plots [1]. It could be computed by Eqn. 4. By incorporating both fixed effects (i.e. climate warming and clipping treatments and their interactions) and random effects (i.e. replicates across different blocks) into the formula $y\sim warming\times clipping+\left(1| block\right)$, LMMs provided a robust and comprehensive assessment of how climate warming and clipping influence PAO diversity and helps identify specific taxonomic groups that are most affected by these experimental treatments.


(4) \begin{equation*} Y= X\beta +Z\ \gamma +\epsilon, \end{equation*}

Where response variable $Y$ denotes the alpha diversity index, design matrix $X$ denotes fixed effects associated with climate change factors (i.e. warming, clipping, and their interaction), matrix $Z$ accounts for random effects (i.e. block structure). The coefficient $\mathrm{\beta}$ represents the effect size of the fixed factors, and $\mathrm{\gamma}$ denotes the random effect size, and $\epsilon$ is an i.i.d normal errors with $\mathbbm{E}\left(\epsilon \right)=0,\sigma \left(\epsilon \right)=1$.

#### PAO network construction and characterizations

Detailed network construction and characterization methodology and formula are provided in the Supplementary Note C and SI Fig. S11. Briefly, to construct the PAO co-occurrence network, we adopted the random matrix theory (RMT)-based methodology as previously described [37, 38, 73] The network was built using Pearson correlation coefficients between OTUs, with nodes representing OTUs and edges representing significant correlations as linkages between OTUs. The network was then partitioned into sub-communities using the Louvain algorithm to show the within-network interactions of PAOs [74]. The role of key taxa (module hubs, connectors, and network hubs) were identified based on within-module connectivity (${Z}_i$) and among-module connectivity (${P}_i$) [2]. The keystone taxa have higher impacts on microbial communities compared to others and are drivers of microbiome structure and functioning [75].

To comprehensively assess the PAO co-occurrence network, we computed two categories of topological metrics: complexity metrics and stability metrics [2]. The complexity metrics (details in Supplementary Note C) include average clustering coefficient ($C$), average degree ($<k>$), centralization of degree (${C}_D$), positive cohesion ($Cohesio{n}_{+}$), connectance ($Conn$), density ($D$), number of edges ($L$), positive edge ratio (${L}_{+}$), efficiency ($E$), geodesic efficiency (${E}_g$), harmonic geodesic distance (${H}_g$), maximal degree (${K}_{max}$), maximal eigenvector centrality (${E}_{max}$), number of network hubs ($Hub$), transitivity ($T$), maximal betweenness (${B}_{max}$), maximal stress (${S}_{max}$), modularity ($M$), number of modules ($Com$), nestedness ($N$), relative modularity ($RM$), and relative nestedness ($RN$). These metrics help quantify the network interconnectedness, structure, and community organization, which provide insights into how taxa interact within the network and the presence of key taxa that drive functional dynamics. They also allow us to understand the hierarchical and modular nature of the PAO network, indicating the strength of interactions within and between sub-communities.

Complementing the complexity metrics, we also computed stability metrics (details in Supplementary Note C) to assess how resilient the network is to perturbations, such as the loss of key taxa or environmental changes. Stability metrics, including robustness ($R$) and vulnerability ($V$), indicate the network capacity to withstand disturbances whereas maintaining functionality. By analyzing these metrics, we can evaluate the network long-term stability and its ability to adapt to environmental stressors. Together, these metrics provide a comprehensive understanding of both the internal organization (complexity) and the external resilience (stability) of the PAO network.

#### Structural equation modeling

In this study, we used SEM to investigate the relationships between experimental treatments, soil chemical profiling, and PAO community, as described previously [1]. Here, the assumption of SEM models was conducted for environmental drivers (SI Fig. S9 & Supplementary Note E). SEM is a powerful statistical technique that enables us to examine the hierarchical relationships between different variables in a complex system. The procedure was repeated until the model showed sufficient fitting, with $P$values of ${\chi}^2$ test $>0.05$ (i.e. the predicted model and observed data are not significantly different) and root mean square error of approximation (RMSE) $<0.08$.

#### Correlation analysis

To investigate the relationships of environmental variables, ecosystem functioning, PAO taxa, and PAO network structures, we computed Pearson and Spearman correlation coefficients and corresponding *P* value using Python3 *SciPy 1.10.1* package.

## Supplementary Material

Supporting_Information_wraf118

## Data Availability

The DNA sequences of the 16S rRNA genes are deposited in NCBI under project accession number PRJNA1063466. Source data are provided within this paper and supporting information.

## References

[1] Wu L, Zhang Y, Guo X. et al. Reduction of microbial diversity in grassland soil is driven by long-term climate warming. *Nat Microbiol* 2022;7:1054–62. 10.1038/s41564-022-01147-3 35697795

[2] Yuan MM, Guo X, Wu L. et al. Climate warming enhances microbial network complexity and stability. *Nat Clim Chang* 2021;11:343–8. 10.1038/s41558-021-00989-9

[3] Cavicchioli R, Ripple WJ, Timmis KN. et al. Scientists’ warning to humanity: microorganisms and climate change. *Nat Rev Microbiol* 2019;17:569–86. 10.1038/s41579-019-0222-5 31213707 PMC7136171

[4] Jansson JK, Hofmockel KS. Soil microbiomes and climate change. *Nat. Rev. Microbiol.* 2020;18:35–46. 10.1038/s41579-019-0265-7 31586158

[5] Zhou Z, Wang C, Luo Y. Meta-analysis of the impacts of global change factors on soil microbial diversity and functionality. *Nat Commun* 2020;11:3072. 10.1038/s41467-020-16881-7 32555185 PMC7300008

[6] Hector A, Bagchi R. Biodiversity and ecosystem multifunctionality. *Nature.* 2007;448:188–90. 10.1038/nature05947 17625564

[7] Filippelli GM . The global phosphorus cycle: past, present, and future. *Elements.* 2008;4:89–95. 10.2113/GSELEMENTS.4.2.89

[8] Melillo JM, Steudler PA, Aber JD. et al. Soil warming and carbon-cycle feedbacks to the climate system. *Science.* 2002;298:2173–6. 10.1126/science.1074153 12481133

[9] Zhou J, Xue K, Xie J. et al. Microbial mediation of carbon-cycle feedbacks to climate warming. *Nat Clim Chang* 2012;2:106–10. 10.1038/nclimate1331

[10] Bradford MA, Wieder WR, Bonan GB. et al. Managing uncertainty in soil carbon feedbacks to climate change. *Nat Clim Chang* 2016;6:751–8. 10.1038/nclimate3071

[11] Greaver TL, Clark CM, Compton JE. et al. Key ecological responses to nitrogen are altered by climate change. *Nat Clim Chang* 2016;6:836–43. 10.1038/nclimate3088

[12] Langhans C, Beusen AHW, Mogollón JM. et al. Phosphorus for sustainable development goal target of doubling smallholder productivity. *Nat Sustain* 2022;5:57–63. 10.1038/s41893-021-00794-4

[13] Tian Y, Shi C, Malo CU. et al. Long-term soil warming decreases microbial phosphorus utilization by increasing abiotic phosphorus sorption and phosphorus losses. *Nat Commun* 2023;14:864. 10.1038/s41467-023-36527-8 36792624 PMC9932148

[14] Chen J, Xu H, Seven J. et al. Microbial phosphorus recycling in soil by intra- and extracellular mechanisms. *ISME Commun* 2024;3:135. 10.1038/s43705-023-00340-7

[15] Duhamel S, Diaz JM, Adams JC. et al. Phosphorus as an integral component of global marine biogeochemistry. *Nat Geosci* 2021;14:359–68. 10.1038/s41561-021-00755-8

[16] Majed N, Wang Z, Baldwin MT. et al. Advancements in phosphorus species profiling and bioavailability assessment with implications for phosphorus sustainability. *Curr Opin Biotechnol* 2025;93:103295. 10.1016/j.copbio.2025.103295 40147310

[17] Akbari A, Wang Z, He P. et al. Unrevealed roles of polyphosphate-accumulating microorganisms. *Microb Biotechnol* 2021;14:82–7. 10.1111/1751-7915.13730 33404187 PMC7888455

[18] Čapek P, Tupá A, Choma M. Exploring polyphosphates in soil: presence, extractability, and contribution to microbial biomass phosphorus. *Biol Fertil Soils* 2024;60:667–80. 10.1007/s00374-024-01829-6

[19] Zhou R, Zhou J, Jia L. et al. Polyphosphate hydrolysis, sorption, and conversion in two different soils. *Eur J Soil Sci* 2023;74:e13341. 10.1111/ejss.13341

[20] Makarov MI, Haumaier L, Zech W. et al. Can ^31^P NMR spectroscopy be used to indicate the origins of soil organic phosphates? *Soil Biol Biochem* 2005;37:15–25. 10.1016/j.soilbio.2004.07.022

[21] Parniske M . Arbuscular mycorrhiza: the mother of plant root endosymbioses. *Nat Rev Microbiol* 2008;6:763–75. 10.1038/nrmicro1987 18794914

[22] Willing CE, Wan J, Yeam JJ. et al. Arbuscular mycorrhizal fungi equalize differences in plant fitness and facilitate plant species coexistence through niche differentiation. *Nat Ecol Evol* 2024;8:2058–71. 10.1038/s41559-024-02526-1 39251818

[23] Carini P, Marsden PJ, Leff JW. et al. Relic DNA is abundant in soil and obscures estimates of soil microbial diversity. *Nat Microbiol* 2016;2:1–6. 10.1038/nmicrobiol.2016.242 27991881

[24] Wang D, He P, Wang Z. et al. Advances in single cell Raman spectroscopy technologies for biological and environmental applications. *Curr Opin Biotechnol* 2020;64:218–29. 10.1016/j.copbio.2020.06.011 32688195

[25] Rinke C, Lee J, Nath N. et al. Obtaining genomes from uncultivated environmental microorganisms using FACS–based single-cell genomics. *Nat Protoc* 2014;9:1038–48. 10.1038/nprot.2014.067 24722403

[26] Majed N, Gu AZ. Application of Raman microscopy for simultaneous and quantitative evaluation of multiple intracellular polymers dynamics functionally relevant to enhanced biological phosphorus removal processes. *Environ Sci Technol* 2010;44:8601–8. 10.1021/es1016526 20949949

[27] Majed N, Matthäus C, Diem M. et al. Evaluation of intracellular polyphosphate dynamics in enhanced biological phosphorus removal process using Raman microscopy. *Environ Sci Technol* 2009;43:5436–42. 10.1021/es900251n 19708378

[28] Hernandez DJ, David AS, Menges ES. et al. Environmental stress destabilizes microbial networks. *ISME J.* 2021;15:1722–34. 10.1038/s41396-020-00882-x 33452480 PMC8163744

[29] Hammarlund SP, Harcombe WR. Refining the stress gradient hypothesis in a microbial community. *Proc Natl Acad Sci USA* 2019;116:15760–2. 10.1073/pnas.1910420116 31320585 PMC6690025

[30] Cadotte MW, Tucker CM. Should environmental filtering be abandoned? *Trends Ecol Evol* 2017;32:429–37. 10.1016/j.tree.2017.03.004 28363350

[31] Zhou J, Ning D. Stochastic community assembly: does it matter in microbial ecology? *Microbiol Mol Biol Rev* 2017;81:10–1128. 10.1128/MMBR.00002-17 PMC5706748 29021219

[32] Louca S, Polz MF, Mazel F. et al. Function and functional redundancy in microbial systems. *Nat Ecol Evol* 2018;2:936–43. 10.1038/s41559-018-0519-1 29662222

[33] García-Palacios P, Gross N, Gaitán J. et al. Climate mediates the biodiversity–ecosystem stability relationship globally. *Proc Natl Acad Sci USA* 2018;115:8400–5. 10.1073/pnas.1800425115 30061405 PMC6099882

[34] White DC, Sutton SD, Ringelberg DB. The genus *Sphingomonas*: physiology and ecology. *Curr Opin Biotechnol* 1996;7:301–6. 10.1016/S0958-1669(96)80034-6 8785434

[35] Barberán A, Bates ST, Casamayor EO. et al. Using network analysis to explore co-occurrence patterns in soil microbial communities. *ISME J.* 2012;6:343–51. 10.1038/ismej.2011.119 21900968 PMC3260507

[36] Shi S, Nuccio EE, Shi ZJ. et al. The interconnected rhizosphere: high network complexity dominates rhizosphere assemblages. *Ecol Lett* 2016;19:926–36. 10.1111/ele.12630 27264635

[37] Xiao N, Zhou A, Kempher ML. et al. Disentangling direct from indirect relationships in association networks. *Proc Natl Acad Sci USA* 2022;119:e2109995119. 10.1073/pnas.2109995119 34992138 PMC8764688

[38] Zhou J, Deng Y, Luo F. et al. Functional molecular ecological networks. *mBio.* 2010;1:e00169–10. 10.1128/mbio.00169-10 PMC2953006 20941329

[39] Wan W, Qin Y, Wu H. et al. Isolation and characterization of phosphorus solubilizing bacteria with multiple phosphorus sources utilizing capability and their potential for lead immobilization in soil. *Front Microbiol* 2020;11:752. 10.3389/fmicb.2020.00752 32390988 PMC7190802

[40] Martín HG, Ivanova N, Kunin V. et al. Metagenomic analysis of two enhanced biological phosphorus removal (EBPR) sludge communities. *Nat Biotechnol* 2006;24:1263–9. 10.1038/nbt1247 16998472

[41] Zhou Y, Pijuan M, Zeng RJ. et al. Could polyphosphate-accumulating organisms (PAOs) be glycogen-accumulating organisms (GAOs)? *Water Res* 2008;42:2361–8. 10.1016/j.watres.2008.01.003 18222522

[42] Li G, Tooker NB, Wang D. et al. Modeling versatile and dynamic anaerobic metabolism for PAOs/GAOs competition using agent-based model and verification via single cell Raman micro-spectroscopy. *Water Res* 2023;245:120540. 10.1016/j.watres.2023.120540 37688851

[43] Bittleston LS . Connecting microbial community assembly and function. *Curr Opin Microbiol* 2024;80:102512. 10.1016/j.mib.2024.102512 39018765

[44] McCann KS . The diversity–stability debate. *Nature.* 2000;405:228–33. 10.1038/35012234 10821283

[45] Wemheuer F, Taylor JA, Daniel R. et al. Tax4Fun2: prediction of habitat-specific functional profiles and functional redundancy based on 16S rRNA gene sequences. *Environ Microbiome* 2020;15:11–2. 10.1186/s40793-020-00358-7 33902725 PMC8067651

[46] Liu XJA, Han S, Frey SD. et al. Microbial responses to long-term warming differ across soil microenvironments. *ISME Commun* 2024;4:ycae051. 10.1093/ismeco/ycae051 38699060 PMC11065356

[47] Womack TM, Crampton JS, Hannah MJ. et al. A positive relationship between functional redundancy and temperature in Cenozoic marine ecosystems. *Science.* 2021;373:1027–9. 10.1126/science.abf8732 34446605

[48] Thakur MP, Tilman D, Purschke O. et al. Climate warming promotes species diversity, but with greater taxonomic redundancy, in complex environments. *Sci Adv* 2017;3:e1700866. 10.1126/sciadv.1700866 28740868 PMC5510977

[49] Ochoa-Hueso R, Collins SL, Delgado-Baquerizo M. et al. Drought consistently alters the composition of soil fungal and bacterial communities in grasslands from two continents. *Glob Change Biol* 2018;24:2818–27. 10.1111/gcb.14113 29505170

[50] Usyskin-Tonne A, Hadar Y, Yermiyahu U. et al. Elevated CO_2_ and nitrate levels increase wheat root-associated bacterial abundance and impact rhizosphere microbial community composition and function. *ISME J* 2021;15:1073–84. 10.1038/s41396-020-00831-8 33208893 PMC8115143

[51] Luo Y, Wan S, Hui D. et al. Acclimatization of soil respiration to warming in a tall grass prairie. *Nature.* 2001;413:622–5. 10.1038/35098065 11675783

[52] Sherry RA, Weng E, Arnone Iii JA. et al. Lagged effects of experimental warming and doubled precipitation on annual and seasonal aboveground biomass production in a tallgrass prairie. *Glob Change Biol* 2008;14:2923–36. 10.1111/j.1365-2486.2008.01703.x

[53] Moebius-Clune BN . Comprehensive Assessment of Soil Health: The Cornell Framework Manual. Ithaca, NY, USA: Cornell University, 2016.

[54] Buyer JS, Sasser M. High throughput phospholipid fatty acid analysis of soils. *Appl Soil Ecol* 2012;61:127–30. 10.1016/j.apsoil.2012.06.005

[55] Tobin KM, McGrath JW, Mullan A. et al. Polyphosphate accumulation by pseudomonas putida CA-3 and other medium-chain-length polyhydroxyalkanoate-accumulating bacteria under aerobic growth conditions. *Appl Environ Microbiol* 2007;73:1383–7. 10.1128/AEM.02007-06 17158616 PMC1828677

[56] Petriglieri F, Singleton C, Peces M. et al. “*Candidatus Dechloromonas phosphoritropha*” and “*Ca. D. Phosphorivorans*”, novel polyphosphate accumulating organisms abundant in wastewater treatment systems. *ISME J* 2021;15:3605–14. 10.1038/s41396-021-01029-2 34155336 PMC8630035

[57] Manzoni S, Ding Y, Warren C. et al. Intracellular storage reduces stoichiometric imbalances in soil microbial biomass–a theoretical exploration. *Front Ecol Evol* 2021;9:714134. 10.3389/fevo.2021.714134

[58] Mason-Jones K, Robinson SL, Veen GF. et al. Microbial storage and its implications for soil ecology. *ISME J.* 2022;16:617–29. 10.1038/s41396-021-01110-w 34593996 PMC8857262

[59] Mason-Jones K, Banfield CC, Dippold MA. Compound-specific ^13^C stable isotope probing confirms synthesis of polyhydroxybutyrate by soil bacteria. *Rapid Commun Mass Spectrom* 2019;33:795–802. 10.1002/rcm.8407 30719792

[60] Chapin Iii FS, Schulze E-D, Mooney HA. The ecology and economics of storage in plants. *Annu Rev Ecol Syst* 1990;21:423–47. 10.1146/annurev.es.21.110190.002231

[61] Ratcliff WC, Kadam SV, Denison RF. Poly-3-hydroxybutyrate (PHB) supports survival and reproduction in starving rhizobia. *FEMS Microbiol Ecol* 2008;65:391–9. 10.1111/j.1574-6941.2008.00544.x 18631180

[62] Mason-Jones K, Breidenbach A, Dyckmans J. et al. Intracellular carbon storage by microorganisms is an overlooked pathway of biomass growth. *Nat Commun* 2023;14:2240. 10.1038/s41467-023-37713-4 37076457 PMC10115882

[63] Yan Y, Lee J, Baldwin M. et al. Discovering polyphosphate and polyhydroxyalkanoate-accumulating organisms across ecosystems: phenotype-targeted genotyping via FACS-sequencing. *bioRxiv*. 2025:2025-03. 10.1101/2025.03.24.644850 41432927

[64] Li G, Wu C, Wang D. et al. Machine learning-based determination of sampling depth for complex environmental systems: case study with single-cell Raman spectroscopy data in EBPR systems. *Environ Sci Technol* 2022;56:13473–84. 10.1021/acs.est.1c08768 36048618

[65] Wang J, Meng S, Lin K. et al. Leveraging single-cell Raman spectroscopy and single-cell sorting for the detection and identification of yeast infections. *Anal Chim Acta* 2023;1239:340658. 10.1016/j.aca.2022.340658 36628751

[66] Wang D, Li Y, Cope HA. et al. Intracellular polyphosphate length characterization in polyphosphate accumulating microorganisms (PAOs): implications in PAO phenotypic diversity and enhanced biological phosphorus removal performance. *Water Res* 2021;206:117726. 10.1016/j.watres.2021.117726 34656820

[67] Movasaghi Z, Rehman S, Rehman IU. Raman spectroscopy of biological tissues. *Appl Spectrosc Rev* 2007;42:493–541. 10.1080/05704920701551530

[68] Li Y, Rahman SM, Li G. et al. The composition and implications of polyphosphate-metal in enhanced biological phosphorus removal systems. *Environ Sci Technol* 2019;53:1536–44. 10.1021/acs.est.8b06827 30589545

[69] Schloss PD, Westcott SL, Ryabin T. et al. Introducing mothur: open-source, platform-independent, community-supported software for describing and comparing microbial communities. *Appl Environ Microbiol* 2009;75:7537–41. 10.1128/AEM.01541-09 19801464 PMC2786419

[70] Douglas GM, Maffei VJ, Zaneveld JR. et al. PICRUSt2 for prediction of metagenome functions. *Nat Biotechnol* 2020;38:685–8. 10.1038/s41587-020-0548-6 32483366 PMC7365738

[71] He P, Son Y, Berkowitz J. et al. Recycled phosphorus bioamendments from wastewater impact rhizomicrobiome and benefit crop growth: sustainability implications at water-food nexus. *Environ Sci Technol* 2025;59:2131–43. 10.1021/acs.est.4c07901. 39841623

[72] Aramaki T, Blanc-Mathieu R, Endo H. et al. KofamKOALA: KEGG ortholog assignment based on profile HMM and adaptive score threshold. *Bioinformatics.* 2020;36:2251–2. 10.1093/bioinformatics/btz859 31742321 PMC7141845

[73] Deng Y, Jiang Y-H, Yang Y. et al. Molecular ecological network analyses. *BMC Bioinformatics* 2012;13:113. 10.1186/1471-2105-13-113 22646978 PMC3428680

[74] Que X, Checconi F, Petrini F. et al. Scalable Community Detection with the Louvain Algorithm. IEEE international parallel and distributed processing symposium 2015; 28–37.

[75] Banerjee S, Schlaeppi K, van der Heijden MGA. Keystone taxa as drivers of microbiome structure and functioning. *Nat. Rev. Microbiol.* 2018;16:567–76. 10.1038/s41579-018-0024-1 29789680

[76] Allen WJ, Sapsford SJ, Dickie IA. Soil sample pooling generates no consistent inference bias: a meta-analysis of 71 plant–soil feedback experiments. *New Phytol* 2021;231:1308–15. 10.1111/nph.17455 33982798

