# Supplementary material for: Climate warming enhances biodiversity and stability of grassland soil phosphorus-cycling microbial communities
Source: ISME J. 2025 Jun 18;19(1):wraf118. doi: 10.1093/ismejo/wraf118 (PMC12366793; doi:10.1093/ismejo/wraf118)
Supplement: Supporting_Information_wraf118 [file supporting_information_wraf118.pdf]

Supplementary Information

Climate Warming Enhances Biodiversity and Stability of Grassland Soil Phosphorus-Cycling Microbial Communities

Zijian Wang[1, 2, †], IL Han[3, †], Jangho Lee[3], Guangyu Li[3], Peisheng He[3], Mathew T. Baldwin[2, 3], Jenny Kao-Kniffin[4], Liyou Wu[5, 6], Jizhong Zhou[5, 6, 7, 8, *], April Z. Gu[2, 3, *]

[1] Department of Biological and Environmental Engineering, College of Agriculture and Life Sciences, Cornell University, Ithaca, NY, USA 14853

[2] Center for Research on Programmable Plant Systems, Cornell University, Ithaca, NY, USA 14853

[3] School of Civil and Environmental Engineering, College of Engineering, Cornell University, Ithaca, NY, USA 14853

[4] School of Integrative Plant Science, Cornell University, Ithaca, NY, USA 14853

[5] Institute for Environmental Genomics, University of Oklahoma, Norman, OK, USA 73019

[6] Department of Microbiology and Plant Biology, University of Oklahoma, Norman, OK, USA 73019

[7] School of Civil Engineering and Environmental Sciences, University of Oklahoma, OK, USA 73019

[8] Earth and Environmental Sciences, Lawrence Berkeley National Laboratory, Berkeley, CA, USA 94720

[*] **Corresponding Author**: April Z. Gu, Department of Civil and Environmental Engineering, Cornell University, 527 College Ave, Ithaca, NY 14850, USA. Email:aprilgu@cornell.edu and Jizhong Zhou, Institute for Environmental Genomics and School of Biological Sciences, University of Oklahoma, 101 David L Boren Blvd, Norman, OK 73019, USA. Email: jzhou@ou.edu

† These authors contributed equally

This file includes:

Supplementary Figure S1-S13

Supplementary Note A, B, C, D, E, and F

Supplementary Table S1-S2

Supplementary Figures

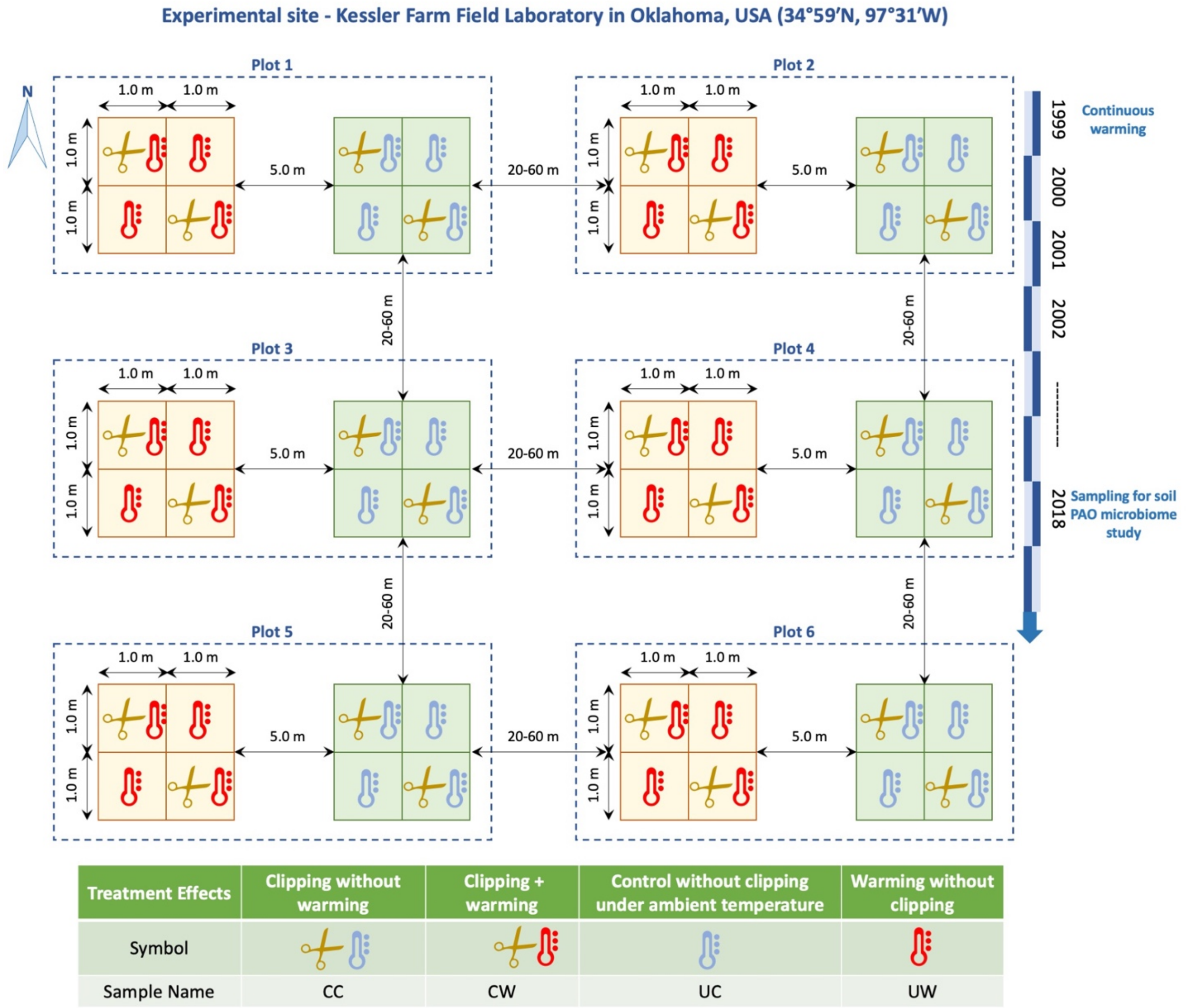

SI Fig. S1| Experimental design and setup at the Kessler Farm Field Laboratory in Oklahoma, USA (34°59′N, 97°31′W). This figure illustrates the layout and treatment schemes of the experimental plots used to study the effects of climate warming and annual clipping on soil bacterial phosphorus-accumulating organisms (PAOs). The experiment was conducted continuously from 1999 to 2018, with soil sampling for PAO biodiversity study occurring in 2018. The field site consists of six plots, each 20 × 60 m, with four subplots per plot. The subplots received one of four treatments: Clipping without warming (CC), Clipping with warming (CW), Control without clipping under ambient temperature (UC), Warming without

clipping (UW). Continuous warming treatments (CW and UW) were applied using infrared heaters that increased soil temperature by approximately 2°C. Clipping was conducted annually to simulate grassland management practices. The symbols and colors represent the different treatment effects applied to each subplot. The treatments were randomly assigned to subplots within each plot to minimize positional effects. The sampling scheme includes soil sampling for the analysis of PAO biodiversity, with each subplot carefully monitored and sampled to ensure consistent data collection. This experimental design allows for the assessment of how long-term climate warming and annual clipping affect soil microbial communities, particularly those involved in phosphorus accumulation.

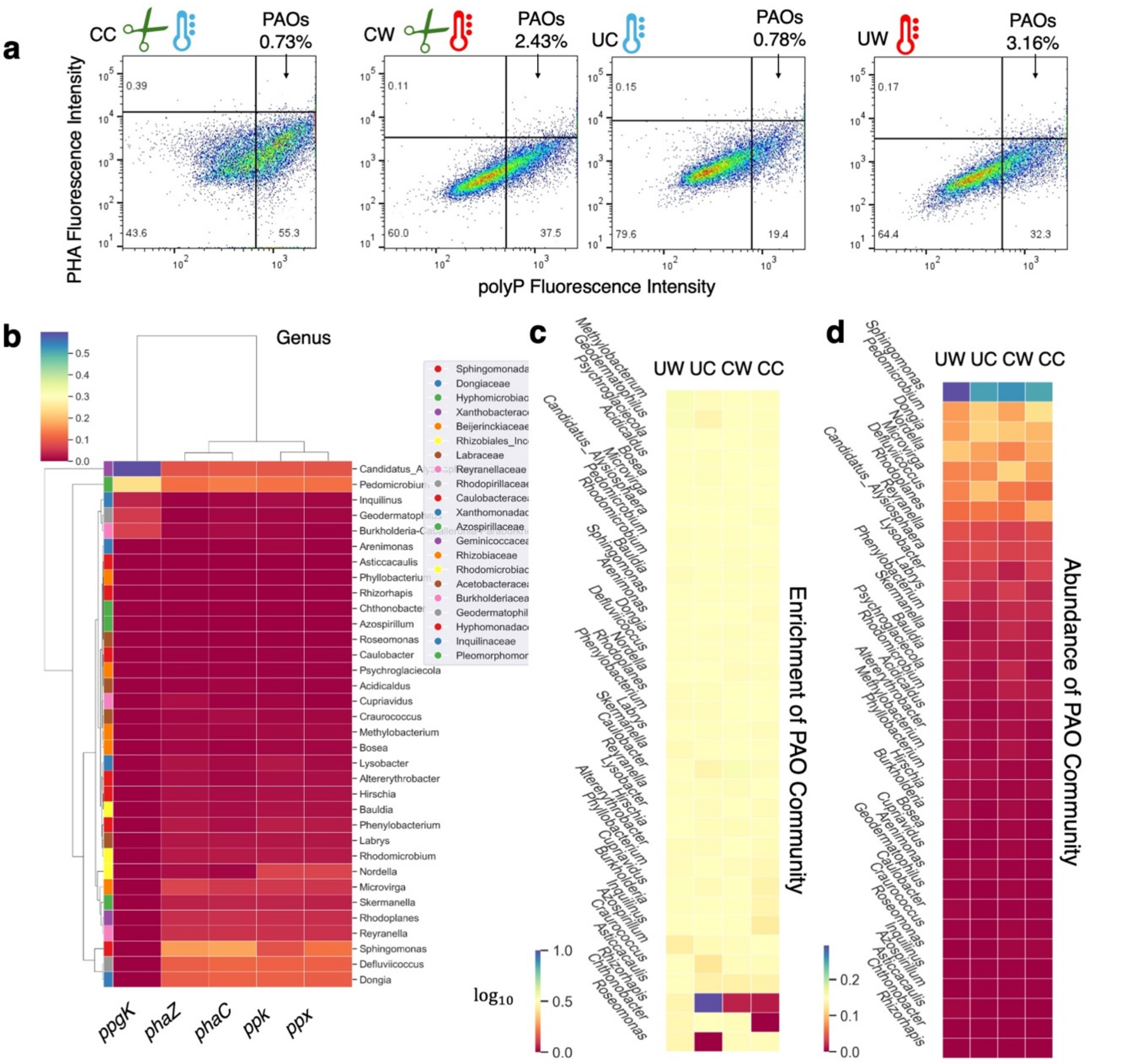

SI Fig. S2 | Fluorescence-activated cell sorting for soil bacterial PAOs. a, Representative flow cytometry plots showing the separation of PAOs based on polyP and PHA fluorescence intensity across different treatment groups, including CC, CW, UC, UW. The percentage of PAOs identified in each treatment group is indicated. **b,** predicted abundance of polyP- (*ppk, ppgK, ppx*) and PHA-related functional genes (*phaC, phaZ*) in FACS-sorted PAOs using PICRUSt2, highlighting PAO taxa with significant metabolic potential for polyP and PHA synthesis and degradation. **c, enrichment of FACS-sorted PAO communities.** The color scale represents log10-normalized enrichment values, with higher enrichment shown in darker shades. **d, Abundance of FACS-sorted PAO communities.** The color scale represents abundance values, with higher abundances shown in blue and green shades. The results show a diverse

distribution of PAO communities across the samples, with some genera exhibiting significantly higher abundances.

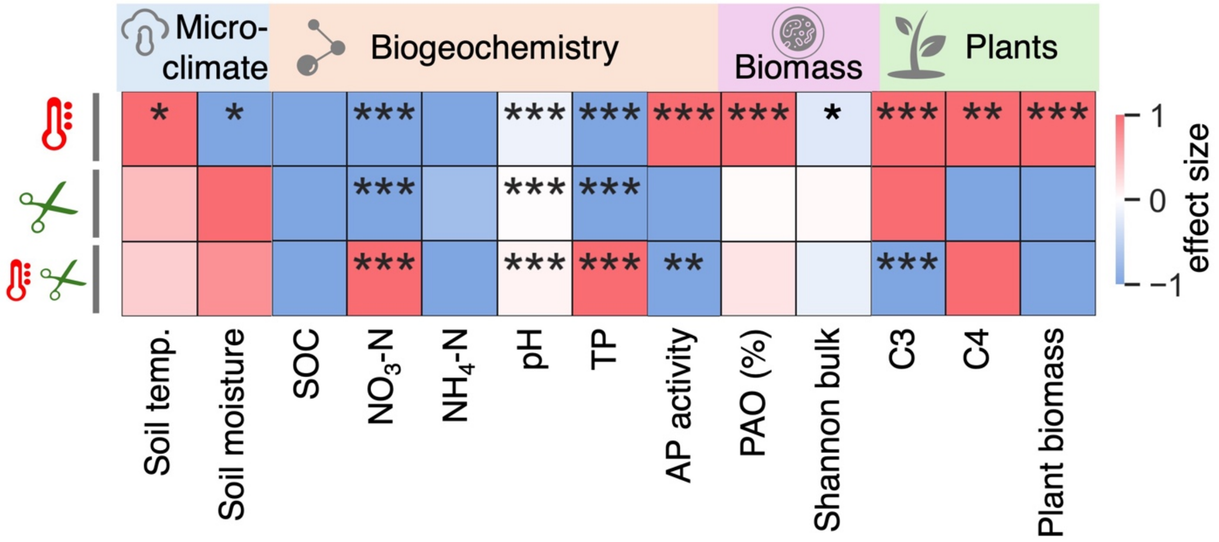

SI Fig. S3 | Treatment effect of climate warming and annual clipping on soil and plant variables by linear mixed-effects models (LMMs). It includes microclimate, biogeochemistry, biomass, and plant variables. Micro-climate includes soil temperature and soil moisture. Biogeochemistry includes soil organic carbon (SOC), soil nitrate (NO_3^-), soil ammonium (NH_4^+), soil pH, soil total phosphorus (TP), soil alkaline phosphatase activity (AP activity). Biomass includes polyphosphate accumulating organisms abundance (PAO%) and bulk bacterial Shannon biodiversity. Plant variables include C3 and C4 biomass and overall plant biomass. The heatmap displays the effect size of each treatment and their interactions, with color gradients indicating the magnitude of the effect. Significance levels are marked as: * *means $P<0.05$, *** *denotes $P<0.01$, **** *denotes $P<0.001$.*

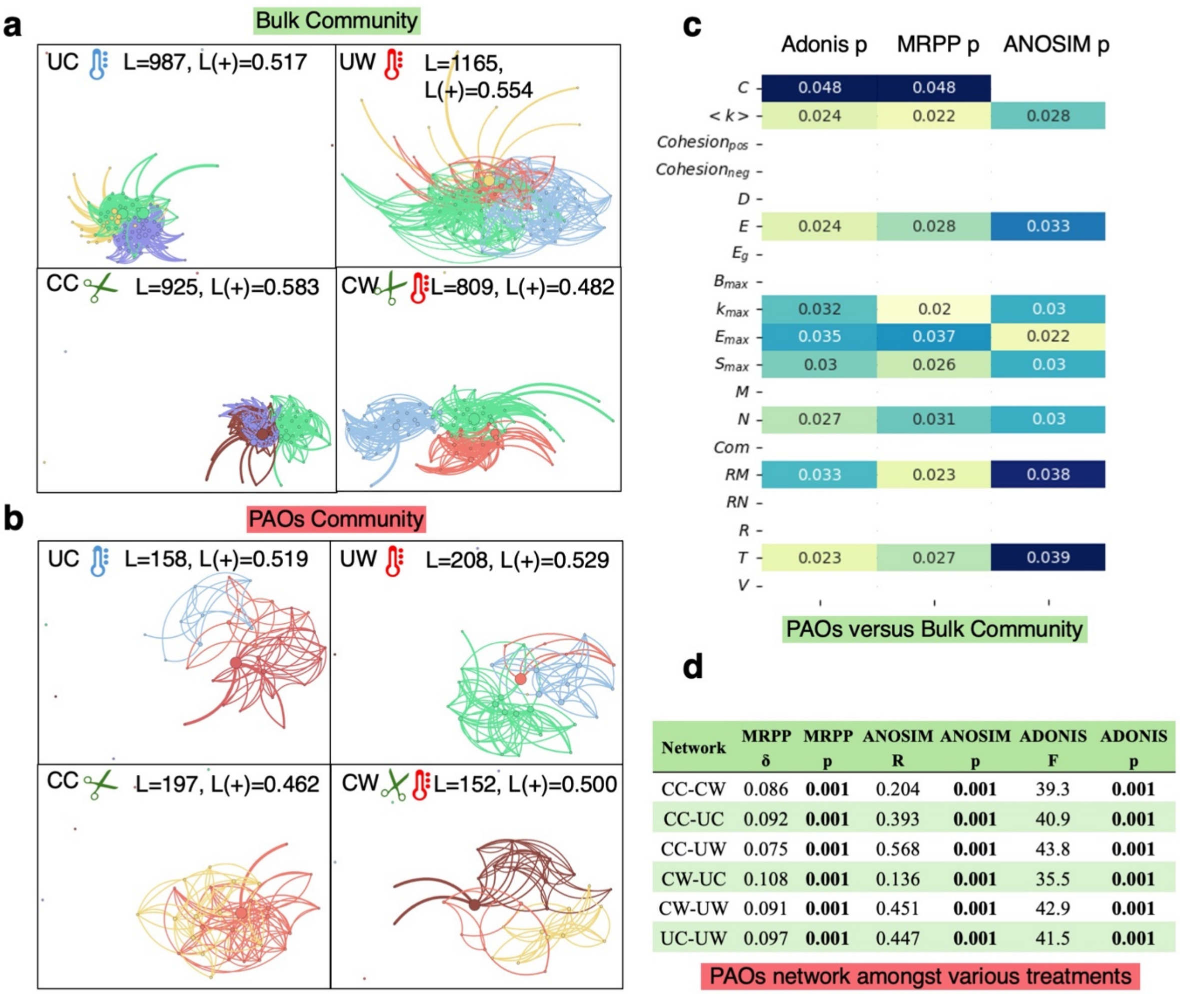

Network	MRPP δ	MRPP p	ANOSIM R	ANOSIM p	ADONIS F	ADONIS p
CC-CW	0.086	**0.001**	0.204	**0.001**	39.3	**0.001**
CC-UC	0.092	**0.001**	0.393	**0.001**	40.9	**0.001**
CC-UW	0.075	**0.001**	0.568	**0.001**	43.8	**0.001**
CW-UC	0.108	**0.001**	0.136	**0.001**	35.5	**0.001**
CW-UW	0.091	**0.001**	0.451	**0.001**	42.9	**0.001**
UC-UW	0.097	**0.001**	0.447	**0.001**	41.5	**0.001**

SI Fig. S4 | Network structures of the PAOs community differ significantly from those of the overall bulk community. a, Network visualizations of the bulk community under different treatment conditions (UC, CC, UW, CW). Each network displays the number of edges (L) and the positive edge ratios (L(+)). **b,** Network visualizations of the PAOs community under the same treatment conditions, with corresponding values of L and L(+). **c,** Statistical comparisons between PAOs and bulk communities using Adonis, MRPP, and ANOSIM tests. **d,** Statistical comparisons between various PAO community under different treatments using Adonis, MRPP, and ANOSIM tests. The colored boxes show the P values < 0.05, indicating significant differences in network structure between PAOs and bulk communities.

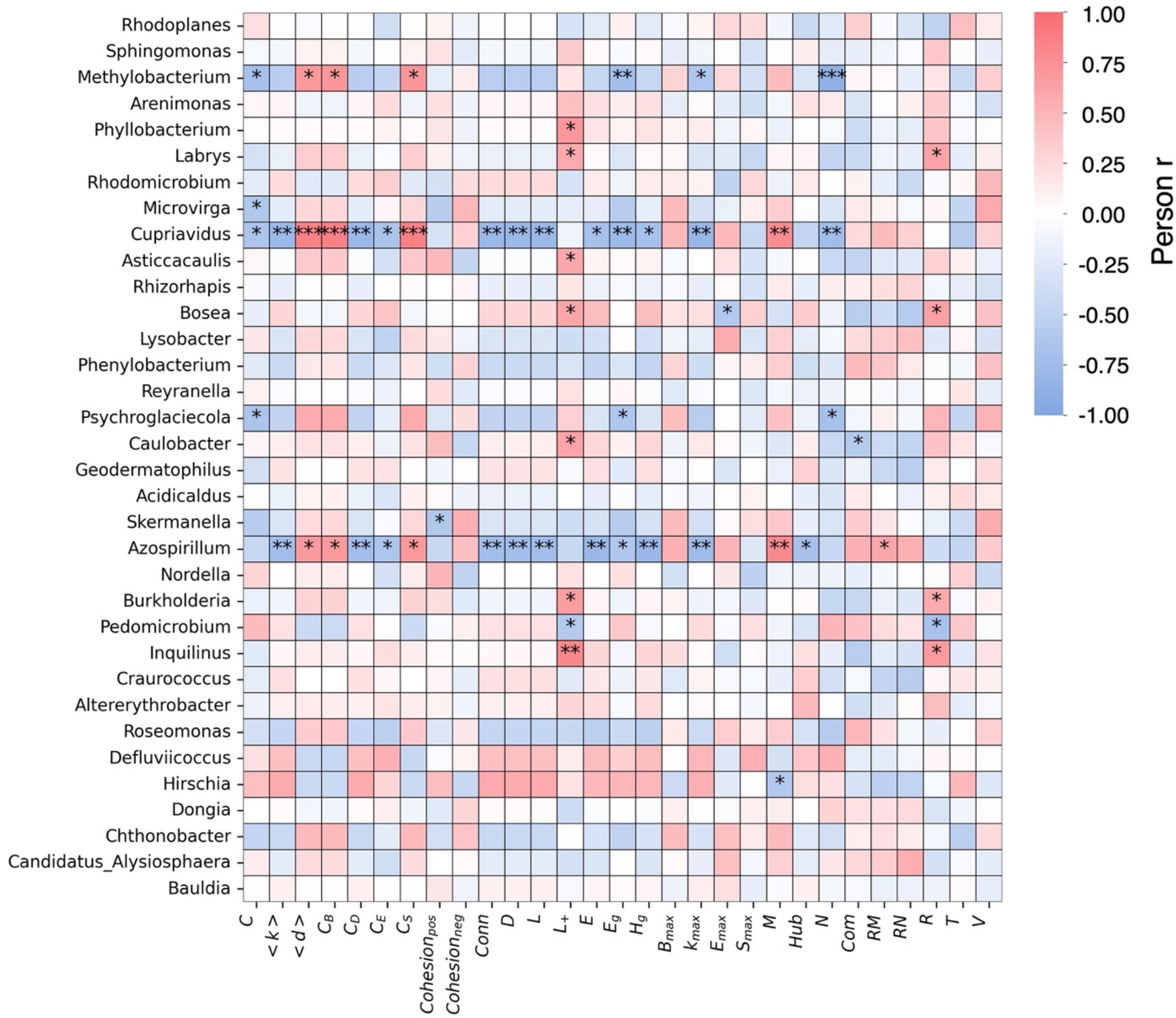

SI Fig. S5 | Pearson correlations between PAO taxa and PAO network complexity and stability metrics. The heatmap illustrates the correlation coefficients (r) between individual PAO genera and various network metrics, indicating the relationship between specific taxa and the complexity and stability of PAO networks. Some genera show strong positive or negative correlations with key network metrics, suggesting their significant roles in network structure and stability. The color scale represents the strength and direction of the correlations, with red indicating positive correlations and blue indicating negative correlations. Significance level: * *means P<0.05, ** denotes P<0.01, *** denotes P<0.001.*

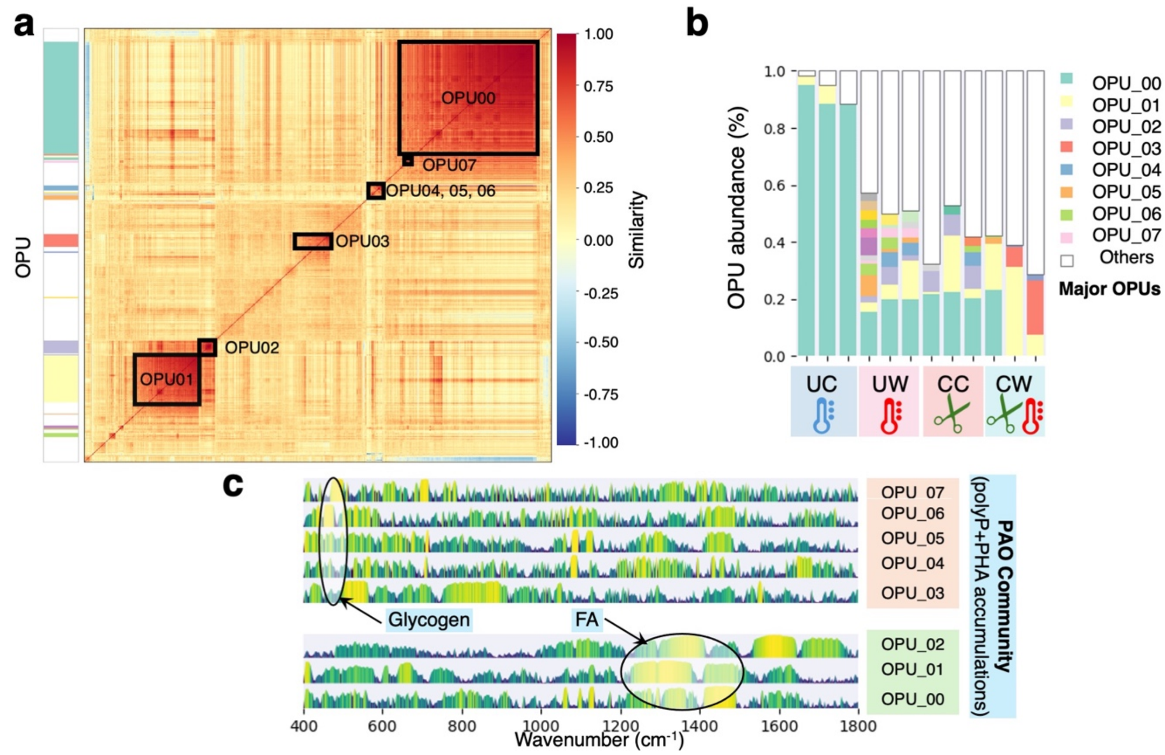

SI Fig. S6 | SCRS-enabled OPU identification, abundance calculation, and molecular ranking within the PAO community. **a,** OPU identification for the PAO community to characterize functional diversity within the PAOs. Major OPUs (OPU00–OPU07) and other OPUs have been identified, with similarity calculated using a cosine-based metric, and hierarchical clustering algorithms used for classification. **b,** The relative abundance of OPUs within different samples (UC, UW, CC, CW), showing the phenotypic composition of the PAO community shifts across conditions. **c,** Fisher feature ranking displaying key molecular signatures within the PAO community. OPU00–OPU02 are enriched in fatty acids (FA), whereas OPU03–OPU07 show higher levels of glycogen. All OPUs contain polyP and PHA, by the definition of PAO community.

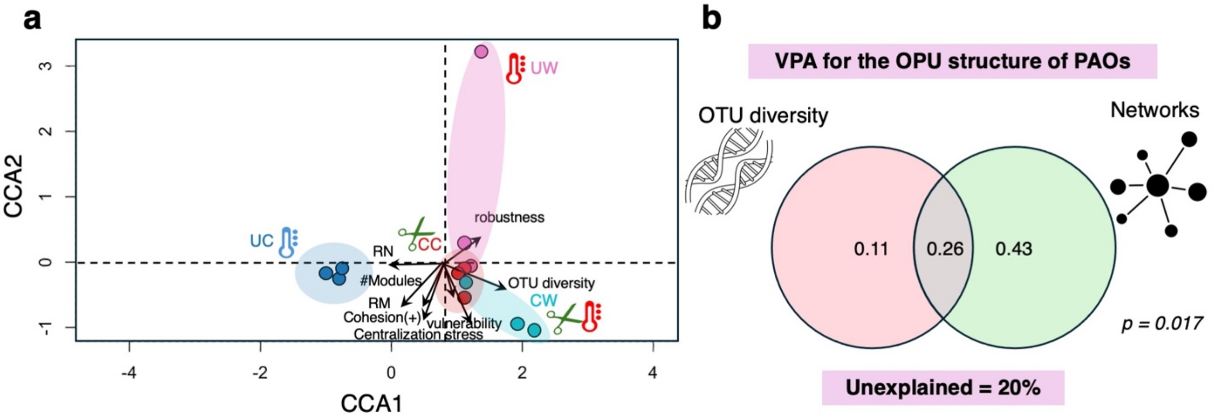

SI Fig. S7 | Functional diversity is well explained by OTU diversity and biotic interactions through CCA-based VPA analysis. a, Canonical Correspondence Analysis (CCA) ordination plot displaying the relationships between PAO community functional composition (OPUs) and taxonomic variables, including OTU diversity and network metrics (representing biotic interactions). The positioning of treatment groups (e.g., UC, UW, CC, CW) indicates how these factors contribute to their distribution and functional diversity. **b,** Variation Partitioning Analysis (VPA) showing that 80% of the variation in OPU structure is explained by OTU diversity and biotic interactions. OTU diversity accounts for 11%, biotic interactions (network metrics) explain 43%, whereas 26% is explained by both, leaving 20% of the variation unexplained ($p < 0.05$).

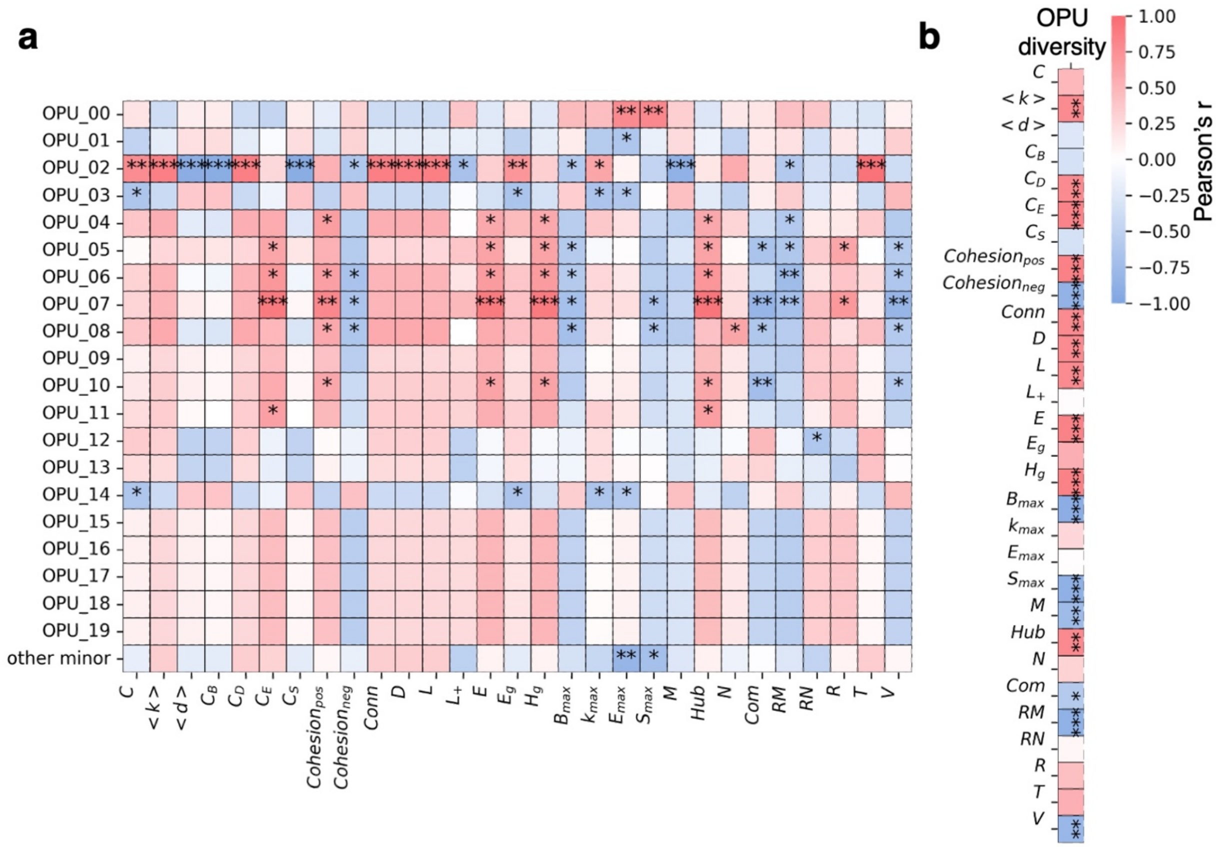

SI Fig. S8 | Correlations between network metrics and OPUs highlight the role of biotic interactions in shaping functional diversity. a, Pearson correlations between network metrics and OPUs. Strong correlations are observed between specific OPUs (e.g., OPU02-07) and network metrics, indicating that biotic interactions significantly influence the distribution and functional traits of these OPUs. **b.** Pearson correlation coefficients between network metrics and OPU diversity. Positive correlations (in red) and negative correlations (in blue) suggest varying degrees of influence of different biotic interactions on functional diversity across the PAO community. Significance level: * *means* $P<0.05$, ** *denotes* $P<0.01$, *** *denotes* $P<0.001$.

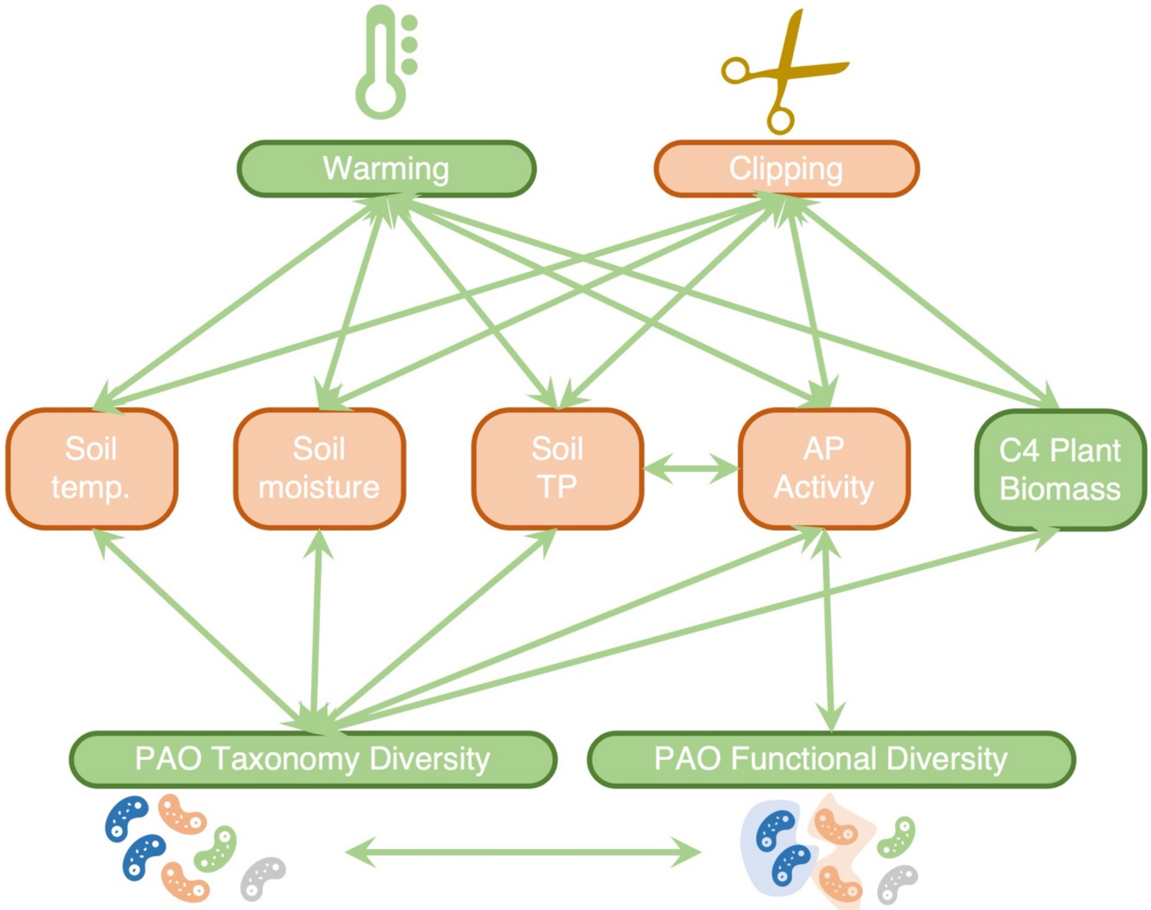

SI Fig. S9 | Assumptions of the Structural Equation Model (SEM) model. Proposed SEM illustrating the hypothesized relationships between climate warming, annual clipping, soil chemical properties and biodiversity of PAO community. The model assumes that climate warming and clipping influence key environmental drivers, including soil temperature, moisture, total phosphorus (TP), and alkaline phosphatase (AP) activity, which in turn affect PAO taxonomic and functional diversity. Additionally, C4 plant biomass is included as a variable influenced by both soil properties and PAO community metrics. Green arrows indicate hypothesized relationships between variables. This initial model will be refined by systematically adding and removing variables and paths to optimize model fit based on observed data (Supplementary Note E).

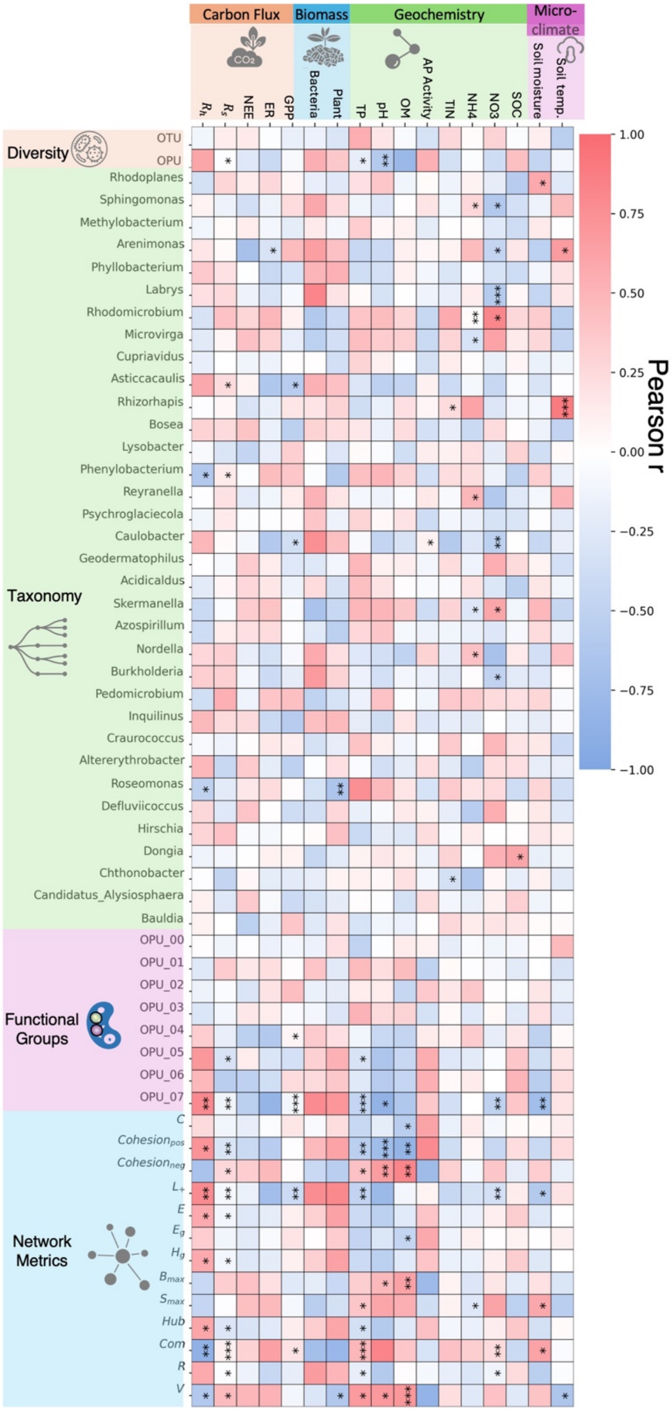

SI Fig. S10 | Correlations between PAO metrics and ecosystem functions, geochemistry, and microbial interactions. Pearson correlation coefficients (r) between key PAO metrics (i.e., diversity, taxonomy, OPU functional groups, and network metrics) and ecosystem functions (i.e., carbon flux, biomass), geochemistry (i.e., soil pH, organic matter (OM), total inorganic nitrogen (TIN), nitrate (NO_3^-), ammonium (NH_4^+), and soil organic carbon (SOC)). Rows represent

different PAO indices, including biodiversity, specific PAO taxa, operational phenotypic units (OPUs), and microbial interactions (network structure metrics). Significance level: * means $P<0.05$, ** denotes $P<0.01$, *** denotes $P<0.001$.

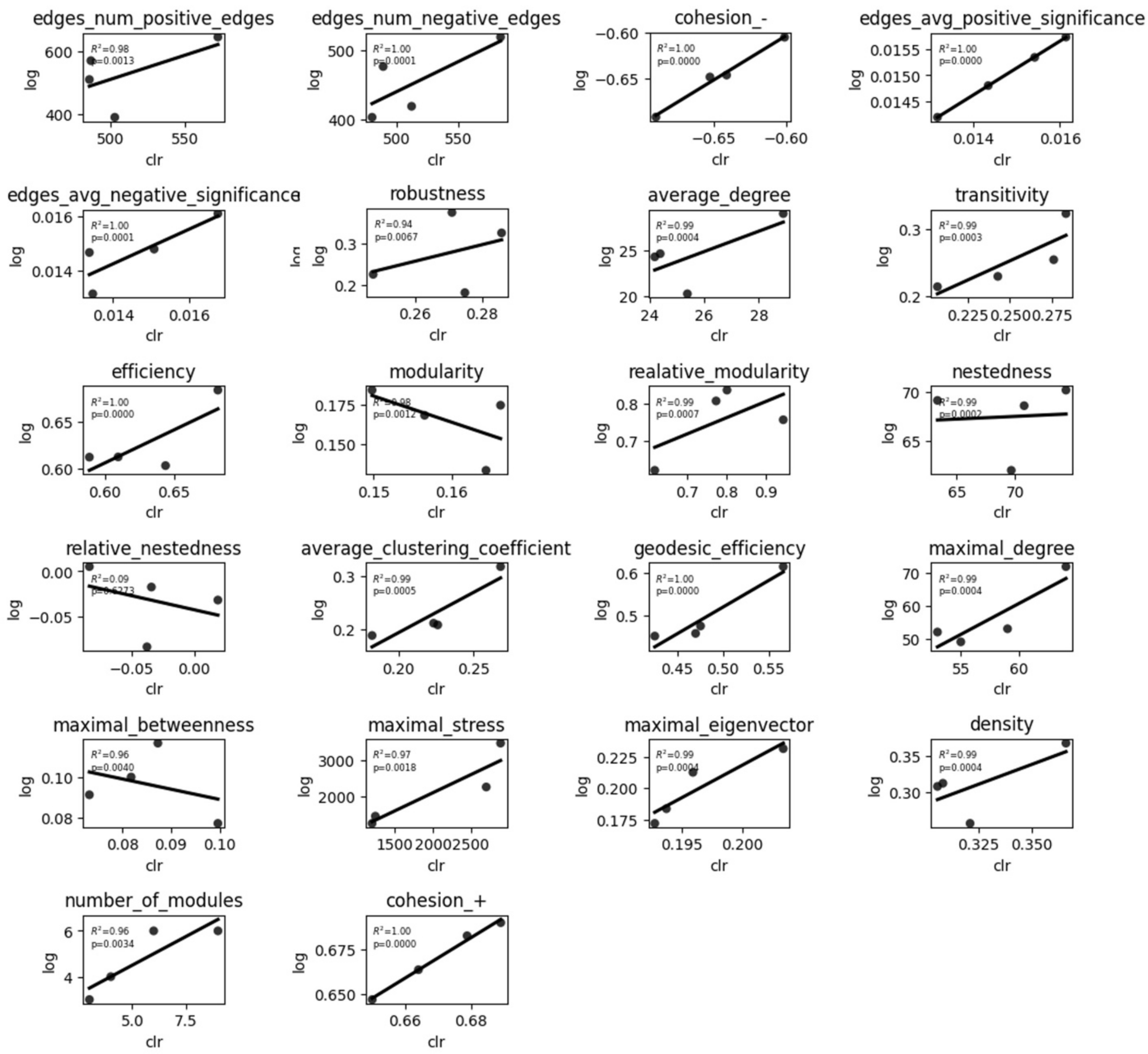

SI Fig. S11 | Correlations of topological metrics of PAO networks under log-transformation and central log-ratio transformation. The analysis reveals strong correlations across various network metrics (n=25) between networks derived from both transformed dataset ($r = 0.94 - 0.99, p < 0.05$), indicating a high degree of similarity between the transformed compositional data. This suggests that the influence of compositional bias on the PAO network structure is negligible.

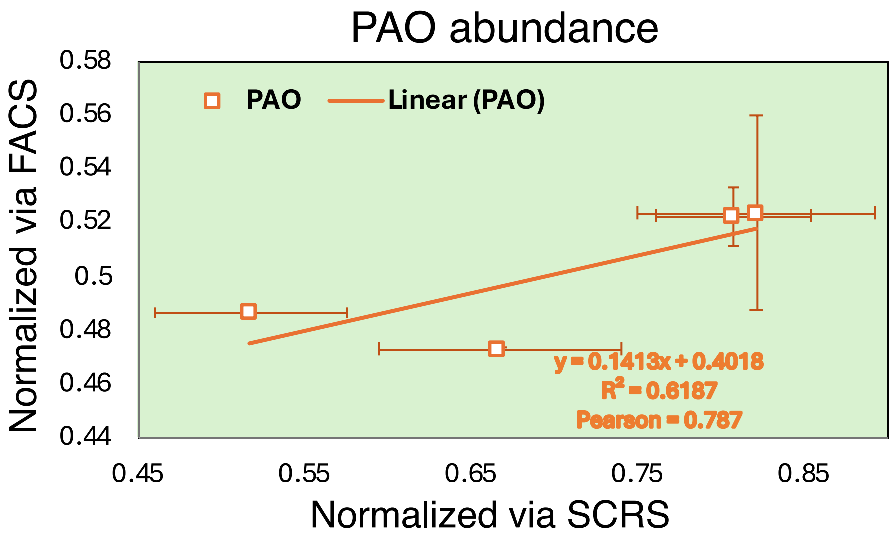

SI Fig. S12 | Validation of PAO quantification using fluorescence-based cytometry compared to single-cell Raman spectroscopy (SCRS). This figure illustrates the relationship between PAO abundance quantified by fluorescence-activated cell sorting (FACS) and single-cell Raman spectroscopy (SCRS). FACS quantifies total cells, including non-bacterial cells, whereas SCRS focuses on bacterial cells. The strong correlation (Pearson $r = 0.787$) between the two methods demonstrates that fluorescence-based cytometry provides a robust and comparable approach to SCRS for quantifying PAOs. The linear regression ($y = 0.141x + 0.4018, R^2 = 0.6167$) further supports the reliability of FACS in PAO quantification.

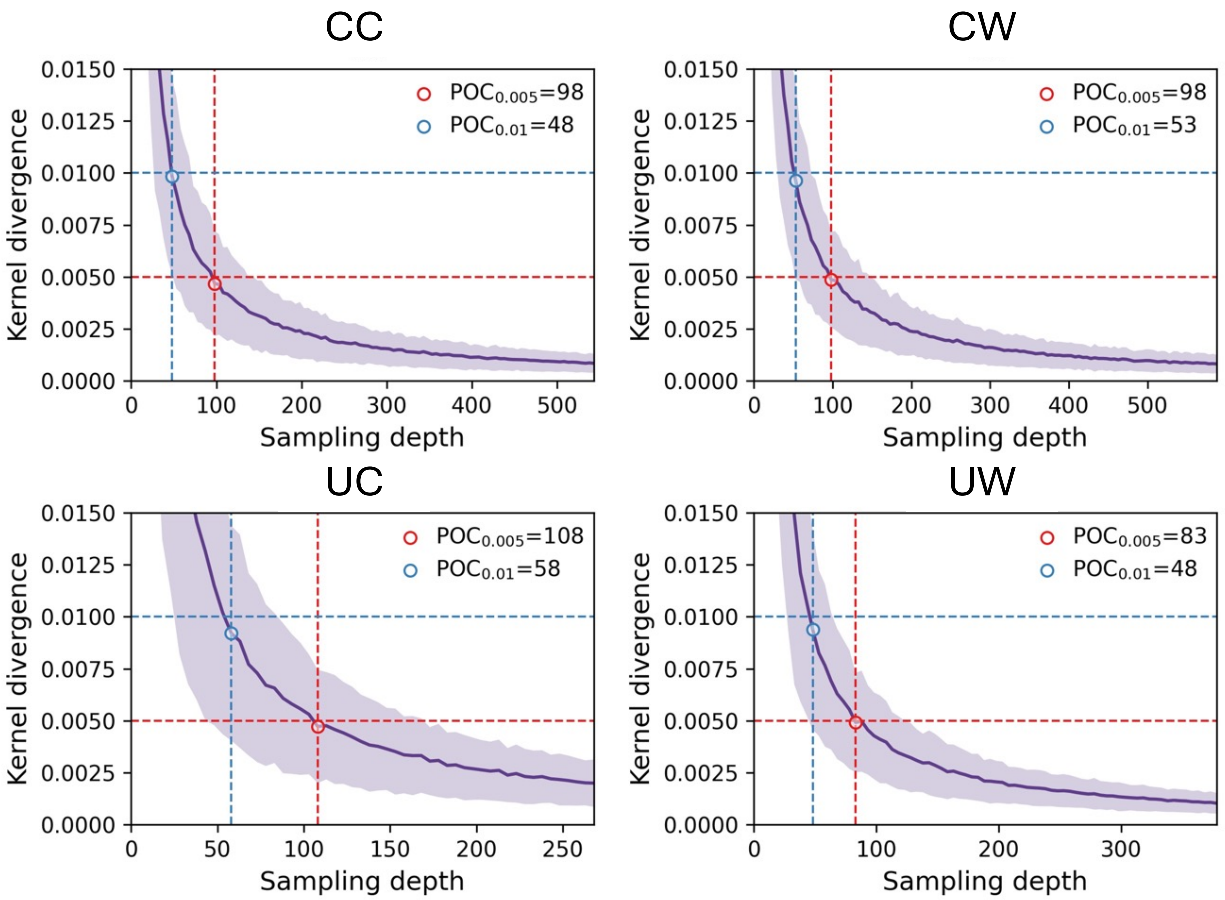

SI Fig. S13 | Single-cell sample size required to characterize PAO community under different treatments. Kernel divergence as a function of sampling depth for different treatment (CC, CW, UC, UW). The purple lines represent the kernel divergence, with shaded regions indicating the standard error. The red and blue dashed lines mark the points of convergence (POC) at different significance thresholds: $POC_{0.005}$ (red circle) corresponds to the sampling depth where the kernel divergence stabilizes at the 0.005 significance level. $POC_{0.01}$ (blue circle) corresponds to the sampling depth where the kernel divergence stabilizes at the 0.01 significance level. Each plot shows the required sampling depth for the specified conditions to achieve stable kernel divergence values. The values of POC provide a measure of how much sampling is required to reach a specified confidence level to characterize a population community (Here, PAO community) for each treatment.

Supplementary Note A: Description of experimental site and setup

The experimental site is located at the Kessler Farm Field Laboratory in Oklahoma, USA (34°59'N, 97°31'W), on a grassland that has remained uncultivated and ungrazed for over 40 years. The vegetation is dominated by C_4 grasses (*Schizachyrium scoparium* and *Sorghastrum nutans*) and C_3 forbs (*Ambrosia psilostachya*, *Solidago rigida*, *and Solidago nemoralis*). The site experiences a mean annual temperature of 16.3°C and an annual precipitation of 914 mm. Soils belong to the Nash-Lucien complex with neutral pH, high available water-holding capacity (~37%), and moderate root penetration potential.[1]

The experiment uses a paired factorial design with warming as the primary factor and clipping as a secondary nested factor (SI Fig. S1). Warming has been imposed continuously since November 21, 1999, via infrared heaters (165 cm x 15 cm; Kalglo Electronics, Bethlehem, PA, USA) delivering 100 W m^{-2} at 1.5 m above the ground. Each 2 m x 2 m plot is subdivided into four 1 m x 1 m subplots, with two diagonal subplots clipped annually at 10 cm height. The clipped materials are removed from the site to further mimic real-world harvesting practices. The other two subplots remain unclipped. Distances between the paired warmed and control plots are maintained at 5 m to avoid heat interference, and plot pairs are spaced 20–60 m apart. The experiment includes four treatments: CC (clipped, control), UC (unclipped, control), CW (clipped, warming), and UW (unclipped, warming).

Temperature increments and moisture content in the top 15 cm of soil were monitored bi-monthly using thermocouples and Time Domain Reflectometry equipment (Soil Moisture Equipment Crop., Santa Barbara, CA, USA). Precipitation data were obtained from the Oklahoma Mesonet Station located ~200 m from the site.

To ensure robust characterization of soil chemical and biological profiles, experiments were designed with sufficient replication across different analyses. For general chemical analyses, flow cytometric counting, and single-cell Raman spectroscopy (SCRS) analysis, six plot-level soil replicates were measured for each treatment condition. As for fluorescence-activate cell sorting (FACS) followed by downstream amplicon sequencing, three replicates were

used by pooling two soil replicates into one group, providing sufficient power for statistical analysis as supported by previous studies.[2, 3]

Supplementary Note B: Treatment effects on soil, plant, and bulk microbial community

Linear mixed-effects models were employed to test the effects of warming and clipping on soil and plant variables, and the bulk microbial community (SI Fig. S3), where effect sizes (β) indicate the magnitudes of treatment impacts.

Soil Microclimate. Warming significantly increased soil temperature ($\beta = 3.97, p < 0.01$) whereas reducing soil moisture ($\beta = -3.30, p < 0.01$), demonstrating the direct impact of climate warming on soil thermal and hydric conditions. In contrast, clipping had no significant effect on soil temperature but slightly increased soil moisture. The increase in soil temperature under warming is expected, as heat input directly raises surface temperatures. The reduction in soil moisture could result from increased evapotranspiration and reduced water retention, typical under warming scenarios. The minor increase in soil moisture under clipping may be due to reduced plant transpiration from lower biomass, leaving more water in the soil. Elevated soil temperature and reduced moisture create harsher conditions for soil microbes, potentially reducing microbial activity, altering species composition, and favoring stress-tolerant species.[4, 5] Drier soils can limit microbial growth, enzyme activity, and organic matter decomposition, impacting nutrient cycling. This environment may favor functional groups, such as polyphosphate-accumulating organisms (PAOs), which are efficient at surviving under nutrient stress.

Soil Geochemistry. Warming induced significant changes in soil biogeochemistry, specifically decreasing both soil pH ($\beta = 0.10, p < 0.001$) and total phosphorus (TP) levels ($\beta = -1.27, p < 0.001$), which are key indicators of nutrient cycling. Additionally, warming significantly increased soil phosphatase activity (AP activity, $\beta = 5.00, p < 0.001$), reflecting an increase in nutrient turnover, and also increased the proportion of polyphosphate-accumulating organisms (PAO%, $\beta = 1.30, p < 0.001$), emphasizing shifts in microbial functionality and nutrient availability under warmer conditions. In contrast, warming had no significant effects on soil organic carbon (SOC), nitrate (NO_3-N), or ammonium (NH_4-N). Clipping, in contrast, had a significant effect only on soil pH ($\beta = 0.05, p < 0.001$), with no detectable impact on SOC, nitrate, ammonium, TP, or AP activity. Clipping also did not

significantly affect PAO%, indicating that nutrient cycling processes are more strongly driven by warming than by vegetation management practices such as clipping.

Warming-induced soil acidification (lower pH) and reduced phosphorus could be due to enhanced microbial activity that accelerates the decomposition of organic matter and the mineralization of phosphorus, depleting available nutrients. The increase in AP activity likely reflects a microbial strategy to cope with phosphorus limitation by producing enzymes to liberate bound phosphorus.[6] Lower pH can inhibit microbial growth and activity for species sensitive to acidic conditions, potentially shifting the community composition toward stress-tolerant microbes.[7] Enhanced AP activity suggests that microbes are responding to nutrient limitations, which could further drive shifts in microbial community composition toward species that are efficient at phosphorus scavenging. The depletion of available phosphorus may favor functional groups with polyphosphate (polyP) accumulation capability, as these microbes can store excess phosphorus in intracellular polyphosphate granules when it is available and use it when external sources are limited. PAOs with the ability to accumulate polyP and PHA (polyhydroxyalkanoates) may be particularly favored under these conditions.

Plant Biomass and Community Composition. Warming significantly increased both C_3 ($\beta = 0.79, p < 0.05$) and C_4 ($\beta = 0.65, p < 0.001$) plant biomass, showing a positive response to elevated temperatures in these functional plant groups. This result underscores the sensitivity of grassland ecosystems to warming, with potential shifts in plant functional group dominance. Clipping, meanwhile, reduced total plant biomass, but its effects on C_3 and C_4 plants were more variable, with clipping tending to favor C_4 plants over C_3 species, although the impact was not as strong as that observed with warming.

The increased biomass of C_3 and C_4 plants under warming could be driven by enhanced photosynthetic activity due to higher temperatures and longer growing seasons, particularly for C_4 species, which are better adapted to warm conditions.[8, 9] The reduction in biomass under clipping results from the removal of above-ground tissues, limiting photosynthesis and carbon allocation to roots. The differential effect of clipping favoring C_4 plants could reflect the fact that C_4 species are more drought and heat-tolerant,[10] allowing them to recover better after biomass removal.

Changes in plant biomass and functional group composition can alter root exudates, which are a key source of carbon for soil microbes. Increased C_4 plant dominance could shift the microbial community toward species that are better adapted to metabolize the carbon compounds released by these plants. Lower overall plant biomass due to clipping could reduce carbon inputs into the soil, limiting microbial growth and reducing microbial biomass, potentially leading to shifts in the structure and function of microbial communities (e.g., a favor of functional groups with PHA accumulations).

Bulk Community and Shannon Diversity. Warming significantly decreased Shannon diversity in the bulk microbial community ($\beta = 0.31, p < 0.01$), indicating a loss of species richness under elevated temperatures. This reduction in diversity is likely linked to the changes observed in soil microclimate and nutrient availability. Clipping, in contrast, did not have a significant effect on Shannon diversity, suggesting that plant biomass removal alone may not exert as strong an influence on microbial community structure compared to the effects of warming.

The decrease in microbial diversity under warming could be due to the environmental filtering effects of higher temperatures, reduced moisture, and altered nutrient availability (such as reduced phosphorus).[5] These environmental stressors may selectively favor microbial species that are more heat- and drought-tolerant with capabilities to adapt to low soil phosphorus and carbon availability, leading to a reduction in overall diversity. The lack of effect of clipping on microbial diversity suggests that the soil microbial community may be more resilient to short-term changes in plant biomass, as long as sufficient carbon inputs remain available through root exudates.

Reduced microbial diversity can have significant implications for ecosystem functioning, as lower diversity may reduce the redundancy and functional capacity of microbial communities. A less diverse microbial community may be less resilient to further environmental changes and may exhibit reduced efficiency in processes like organic matter decomposition and nutrient cycling, which could ultimately affect soil health and plant productivity. However, it remains unclear how specific functional groups are affected. Some functional groups, such as PAOs with the ability to accumulate polyP and PHA, may adapt better to these conditions, maintaining or even increasing their abundance despite the overall loss in diversity. This could lead to shifts in

functional composition, allowing certain processes, like phosphorus and carbon storage, to continue under altered environmental conditions.

Supplementary Note C: Bacterial PAO network construction and characterization

1. **PAO Network Construction**

To construct the PAO network, we adopted random matrix theory (RMT)-based methodology as previously described [11-13] Briefly, we firstly computed a correlation matrix $\rho \in \mathbb{R}^{d_o \times d_0}$ of the d_0 OTUs across n bio-samples, which was used to construct an original graph G_0 for each cutoff value v. The cutoff value is to filter out the absolute value of correlation coefficients $|\rho|$ that is smaller than v. The cutoff value v ranges from 1 to 0 with step size s because $|\rho| \in [0,1]$. Subsequently, n random graphs $G_i, i \in [1,2,\ldots,n]$ were generated for each cutoff value v using *Maslov-Sneppen* algorithm by randomly swapping edges in G_0 while keeping the number of vertices and edges unchanged.[14] Then, d graph metrics were computed for each graph $f: G \rightarrow \mathbb{R}^d$, such as generalized degree coefficients. Finally, multivariate statistical testing *Hotelling's* T-squared distribution was performed on the metric vector of original graph $f(G_0) \in \mathbb{R}^d$ and random graph matrix $f(G_1,\ldots,G_n) \in \mathbb{R}^{d \times n}$, thus producing a statistics value and corresponding p value.[15] Because multivariate metrics are used in statistical testing, false discovery rate (FDR) was performed to correct p value.[16] Therefore, in a decreasing order of cutoff v_l, we can obtain a sequence of p_l^{FDR} value, where $l \in \left[1, \min\left(50, \frac{1}{s}\right)\right]$. Meanwhile, the eigenvalue spacing e distribution $P(e)$ of the correlation matrix ρ was constructed for each cutoff value v, which would be statistically tested to follow Poisson distribution or Gaussian orthogonal ensemble statistics. The optimal graphs G_l can be determined for $p_l^{FDR} < 0.05$ and $P(e)_l$ follows Poisson distribution. Specifically, in this study, we are using Pearson correlation coefficients for correlation matrix ρ, step size $s = 0.01$, and random graph $n = 100$. We computed $d = 4$ metrics for each graph, including generalized degree coefficients, clustering coefficients, transitivity, average shortest path length.

Once the graph G_l has been constructed, p values for edges of G_l were adjusted with FDR and only the edge with significance $p^{FDR} < 0.05$ were kept. Specifically, PAO network was constructed where nodes represent the OTUs and edges represent significant correlations between them (edges were colored either green or red to depict positive or negative correlations,

respectively). Subsequently, the network was partitioned into sub-communities using the Louvain algorithm to show the within-network interactions of PAOs.[17]

2. PAO Network are Scale-free

The PAO networks under all treatment conditions (CC, UC, CW, UW) exhibit scale-free characteristics (Table S1), as indicated by the power-law distribution of node connectivity, with a few highly connected hub species and many others with fewer connections. This suggests that certain PAO species play a key role in maintaining network structure and functionality, such as P cycling. Under both warming and clipping, the networks continue to follow a scale-free pattern, with some variation in the degree of reliance on these hub species. These results indicate that while the network remains scale-free under climate changes, a random loss of key hub species may impair the PAO network stability.

3. PAO Network Characterization

To further identify the PAO network hubs, we adopted a previously reported methodology that requires the computation of within-module connectivity (Z_i) and among-module connectivity (P_i).[18] Specifically, module hubs ($Z_i \geq 2.5, P_i < 0.62$), connectors ($Z_i < 2.5, P_i \geq 0.62$), and network hubs ($Z_i \geq 2.5, P_i \geq 0.62$) could be identified as keystone taxa within the PAO network.[18, 19] The keystone taxa have higher impacts on microbial communities compared to others and are drivers of microbiome structure and functioning.[20]

Moreover, various network metrics were calculated to characterize the PAO co-occurrence network, with specific definitions and meaning to PAO networks in Table S2. Complexity metrics assess the structural organization, such as connectivity, modularity, and interactions between taxa, which help reveal the network overall functionality and robustness. Metrics like clustering coefficient, degree centralization, and modularity allow us to understand the hierarchical nature and community structure of the network. Stability metrics like robustness and vulnerability were computed to evaluate the network resilience to perturbations and its ability to maintain functionality despite the loss of nodes or links. Together, these metrics provide a comprehensive view of how the network is organized and how well it can withstand environmental changes.

Complexity Metrics

- **Average Clustering Coefficient ($\boldsymbol{C}$)**

$$C = \frac{1}{n}\sum_{i=1}^{n}\frac{2e_i}{k_i(k_i-1)},$$

Where e_i is the number of edges between the neighbors of node i, and k_i is the degree of node i.

- **Average Degree ($< \boldsymbol{k} >$)**

$< k > = \frac{2L}{n},$

Where L is the total number of edges, and n is the number of nodes.

- **Centralization of Degree ($\boldsymbol{C_D}$)**

$$C_D = \frac{\sum_{i=1}^{n}(k_{max}-k_i)}{(n-1)(n-2)},$$

Where k_{max} the maximum degree in the network, and k_i is the degree of node i.

- **Cohesion ($\boldsymbol{Cohesion_+}$ and $\boldsymbol{Cohesion_-}$)**

$$\text{Cohesion}_{+/-} = \sum_{i=1}^{m}\text{abundance}_i \times \text{connectedness}_{i,+/-},$$

Where m is the total number of taxa in the community, $abundance_i$ is the relative abundance of taxon i, and $connectedness_i$ is the null model-corrected pairwise correlation for taxon i.

- **Connectance ($\boldsymbol{Conn}$)**

$$\text{Connectance} = \frac{L}{n(n-1)},$$

Where L is the number of edges, and $n(n-1)$is the total number of possible edges.

- **Density ($\boldsymbol{D}$)**

$$D = \frac{2L}{n(n-1)},$$

Where L is the number of edges, and n is the number of nodes.

- **Number of Edges (L)**

The total number of edges in the network by counting the edges once the network is constructed.

- **Positive Edge Ratio (L_+)**

$$L_+ = \frac{\text{Number of positive edges}}{L},$$

Where L is the number of edges, which measures proportion of positive (cooperative) interactions.

- **Efficiency (E)**

$$E = \frac{1}{n(n-1)} \sum_{i \neq j} \frac{1}{d(i,j)},$$

Where $d(i,j)$ is the shortest path between nodes i and j.

- **Geodesic Efficiency (E_g)**

$$E_g = \frac{1}{\sum_{i,j} d(i,j)},$$

Where $d(i,j)$ is the shortest path between nodes i and j, which measures global efficiency in terms of path lengths between nodes.

- **Harmonic Geodesic Distance (H_g)**

$$H_g = \frac{1}{n} \sum_{i \neq j} \frac{1}{d(i,j)}$$

This metric evaluates the efficiency of shortest paths across the network.

- **Maximal Degree (K_{max})**

$$K_{max} = \max(k_i)$$

The highest degree of any node in the network.

- **Maximal Eigenvector Centrality (E_{max})**

$$A\boldsymbol{x} = \lambda \boldsymbol{x}$$

Where λ is the largest eigenvalue of the adjacency matrix A, and $\boldsymbol{x}$ is the corresponding eigenvector.

- **Number of Network Hubs ($\boldsymbol{Hub}$)**

No standard equation—this is typically determined based on thresholds for high centrality or degree values.

- **Transitivity ($\boldsymbol{T}$)**

$$T = \frac{3 \times \text{Number of triangles}}{\text{Number of connected triples of vertices}}$$

Measures the likelihood of nodes forming tightly knit groups.

- **Maximal Betweenness ($\boldsymbol{B_{max}}$)**

$$B(v) = \sum_{s \neq v \neq t} \frac{\sigma(s, t|v)}{\sigma(s, t)},$$

Where $\sigma(s, t|v)$ is the number of shortest paths from node s to node t that pass through node t, and $\sigma(s, t)$ is the total number of shortest paths.

- **Maximal Stress ($\boldsymbol{S_{max}}$)**

$$S(v) = \sum_{s \neq v \neq t} \sigma(s, t|v),$$

Indicates the control a node exerts over information flow.

- **Modularity ($\boldsymbol{M}$)**

$$M = \frac{1}{2L} \sum_{i,j} \left[A_{ij} - \frac{k_i k_j}{2L} \right] \delta(c_i, c_j),$$

Measures how well the network is divided into modules.

- **Number of Modules ($\boldsymbol{Com}$)**

This metric is determined by counting the number of distinct community structures (modules) within the network.

- **Relative Modularity ($\boldsymbol{RM}$)**

$$RM = \frac{M - \overline{M_r}}{\overline{M_r}},$$

Where M is the modularity of the empirical network, and $\overline{M_r}$ is the mean modularity of random networks.

- **Relative Nestedness ($\boldsymbol{RN}$)**

$$RN = \frac{N - \overline{N_r}}{\overline{N_r}},$$

Where N is the nestedness of the empirical network, and $\overline{N_r}$ is the mean nestedness of random networks.

Stability Metrics

- **Robustness ($\boldsymbol{R}$)**

$$\mathrm{wMIS}_i = \frac{\sum_{j \neq i} b_j s_{ij}}{\sum_{j \neq i} b_j},$$

Where b_j is the relative abundance of species j, and s_{ij} is the association strength between species i and j. The robustness is measured as the proportion of nodes remaining after random or targeted node removal.

- **Vulnerability ($\boldsymbol{V}$)**

$$\max\left(\frac{\mathrm{E} - \mathrm{E_i}}{\mathrm{E}}\right)$$

Where E is the global efficiency of the network, and E_i is the global efficiency after removing node i and its links.

4. **Transformation Bias of PAO Networks are Negligible**

The decision to use log transformation instead of central log-ratio (CLR) transformation is based on the negligible bias introduced by either method, as shown by the strong correlations between network metrics derived from both transformations. Figure S11 demonstrates that topological metrics of PAO networks under log and CLR transformations are highly correlated (r = 0.94-1.0,

$P<0.05$) across 25 network metrics, indicating a high degree of similarity between the networks derived from both transformations.

This strong correlation suggests that the influence of compositional bias on the PAO network structure is minimal regardless of the transformation applied. Given that log transformation yields similar results to CLR transformation while being computationally efficient, it was chosen for this analysis. The minimal difference between the transformations supports the conclusion that log transformation is an appropriate choice, providing accurate insights into the PAO network topology without significant distortion from compositional effects.

Supplementary Note D: Single-cell Raman Spectroscopy-enabled functional characterization of PAOs

1. SCRS as a robust and complementary single-cell technique compared to FACS

Single-cell Raman Spectroscopy (SCRS) is a high-sensitive, label-free, and non-invasive technique that enables direct biochemical characterization of individual cells based on their unique Raman spectral signatures.[21] In the context of polyphosphate-accumulating organisms (PAOs), SCRS complements fluorescence-activated cell sorting (FACS) by providing a full-spectrum molecular characterization of PAOs. While FACS selectively sorts cells based on pre-defined markers like polyphosphate (polyP) and polyhydroxyalkanoates (PHA), SCRS can captures a broader spectrum of molecular information, including these markers and other intracellular metabolites, through vibrational signatures.

Such comprehensive profiling enables advanced downstream analyses of the PAO community. It allows for the study of molecular dynamics under different environmental or treatment conditions, Operational Phenotypic Unit (OPU) analysis to group cells by functional traits, and the quantification of the functional diversity of the PAO community. By providing a detailed understanding of intracellular composition, SCRS goes beyond sorting, facilitating a deeper exploration of the functionality of PAO community. Moreover, mutual validation between SCRS and fluorescence-based cytometry demonstrates that both methods provide robust and comparable PAO quantification, highlighting their complementary strengths (SI Fig. S12).

2. Definition of Operational Phenotypic Units

The introduction of Operational Phenotypic Units (OPUs) is motivated by Operational Taxonomic Units (OTUs),[22] which are widely employed in taxonomic classification and biodiversity studies based on genetic sequence similarity. While OTUs provide a robust framework for categorizing microbial communities, they inherently focus on genetic relationships and overlooks functional diversity within taxa. In the context of polyphosphate-accumulating organisms (PAOs), where metabolic traits such as polyphosphate (polyP) and polyhydroxyalkanoates (PHA) accumulation are critical, reliance solely on taxonomic units may obscure key functional phenotypes that drive ecosystem processes. Thus, a complementary approach, centered on phenotypic rather than genetic traits, is warranted.

Hence, OPUs are defined as clusters of individual cells that exhibit similar functional characteristics, as determined by clustering algorithms and Single-cell Raman Spectroscopy (SCRS) profiles. These profiles capture the unique biochemical signatures of cells, reflecting the abundance of intracellular compounds such as polyP and PHA. In contrast to OTUs, which cluster organisms based on genetic sequences, OPUs categorize cells by their metabolic phenotypes, allowing for a more direct assessment of their functional roles within microbial communities. This phenotypic approach is particularly advantageous for studying PAOs, where functional activity is a primary determinant of ecological contribution.

3. OPU-based advanced analysis

The application of OPUs enables a more nuanced understanding of microbial community function, extending beyond taxonomic diversity to characterize metabolic diversity. By employing OPUs, researchers can (1) map functional biodiversity within PAO communities and (2) identifying the distinctive molecular characteristic of each OPU that contribute to key processes such as nutrient cycling and energy storage. This functional perspective provides a deeper insight into the ecological roles of PAOs and allows for a more comprehensive understanding of microbial dynamics than is possible through OTU-based analyses alone.

3.1 Computation of functional diversity

To assess the functional diversity within the PAO community, Shannon entropy (H') can be calculated based on the abundance of OPUs identified from the SCRS profiles. Shannon entropy is a widely used index to measure diversity, incorporating both the richness (number of unique OPUs) and evenness (distribution of OPUs across the community). The calculation of Shannon entropy follows the formula:

$$H' = -\sum_{i=1}^{n} p_i \, log(p_i)$$

Where:

- p_i is the proportion of individuals belonging to OPU i.
- n is the total number of OPUs identified in the community.

This index provides a quantitative measure of the functional diversity, offering a clearer understanding of how different treatment effects will influence the PAO functional diversity.

3.2 Feature ranking to identify distinctive molecules for OPUs

Because Raman spectroscopy provides full-spectrum characterization, after identifying the OPUs, feature ranking is used to identify the key differentiating Raman spectral features amongst OPUs corresponding to important intracellular molecules such as fatty acids, glycogen, polyP, and PHA. Algorithms such as Fisher's ranking[23] or other statistical methods are employed to rank the Raman wavenumbers based on their contribution to the differentiation of OPUs. This ranking allows us to determine which specific spectral features (wavenumbers) correspond to the most critical molecular metabolites, enabling the identification of key molecular signatures that define the functional differences among the OPUs.

This approach enhances the understanding of the metabolic diversity within the PAO community by focusing on the specific biochemical traits that drive functional variation across OPUs. It also provides a foundation for further studies on how these functional traits influence the ecological roles of PAOs under different environmental conditions.

4. Single-cell sample size is sufficient to characterize PAO community

To ensure accurate characterization of the PAO community, we performed SCRS on a statistically significant number of single cells. The sample size was computed based on prior studies and kernel divergence-based algorithms to capture the phenotypic diversity within the community.[24] The SCRS profiles obtained were robust enough to characterize PAO community for each sample (SI Fig. S13), and to differentiate between distinct OPUs, allowing for comprehensive coverage of the functional landscape. Moreover, under warming or clipping conditions (UW, CC, CW), fewer single cells were required to accurately characterize the community compared to the control (UC), suggesting that the community became more specialized or homogeneous in response to these environmental stressors. This sampling size was sufficient to draw meaningful conclusions regarding the metabolic roles of PAOs in polyP and PHA accumulation.

Supplementary Note E: Linkage of PAO metrics and ecosystem functioning

1. Assumption of structural equation modeling

In this study, Structural Equation Modeling (SEM) was employed to explore the relationships between experimental treatments, soil chemical properties, PAO community metrics, and ecosystem functioning, following the framework as previously described.[5] SEM is a robust statistical method that allows us to analyze complex, hierarchical relationships by modeling the interactions between multiple variables simultaneously. In this context, SEM was used to investigate how climate warming, soil microclimate variables (e.g., temperature, moisture), and soil phosphorus availability influenced PAO taxonomic biodiversity and functional diversity.

We firstly proposed a theoretical model based on prior knowledge on PAOs (SI Fig.S9), with environmental factors such as soil temperature, moisture, total phosphorus (TP), and alkaline phosphatase (AP) activity hypothesized as primary drivers influencing PAO diversity. In the initial model, all relevant environmental and PAO community metrics were included as paths.

Next, variables or paths were systematically added or removed based on the statistical significance of their contributions and ecological relevance. This iterative adjustment process was designed to improve the model fit and ensure parsimony. Paths with non-significant coefficients were removed unless they were crucial for maintaining biological integrity based on prior knowledge. Conversely, new paths were added if initial results indicated overlooked interactions or indirect effects.

Each iteration was evaluated using key fit indices such as the chi-square test ($P > 0.05$ indicating no significant difference between the assumption model and observed data), root mean square error of approximation ($RMSEA < 0.08$), and comparative fit index ($CFI > 0.90$). The adjustments continued until the model demonstrated sufficient fit to the observed data, balancing both statistical rigor and biological realism.

Through this process, we were able to identify the most influential environmental drivers and their pathways affecting PAO diversity, network complexity, and ecosystem functioning under warming conditions. The final SEM highlights how environmental filtering affects the taxonomic biodiversity of the PAO community.

This approach allowed us to unravel the direct and indirect pathways through which environmental drivers influenced PAO diversity, highlighting the role of environmental filtering in shaping the response of microbial community to climate warming.

2. Relationships of PAOs and soil geochemistry and ecosystem functioning

The correlation analysis reveals significant associations between PAO diversity, specific PAO taxa, functional groups, and ecosystem functioning, such as carbon flux (SI Fig. S10). Here, we discuss two perspectives of PAO roles, including in soil carbon cycling and phosphorus cycling.

2.1 Role of PAO in Soil Carbon Cycling

PAOs act primarily as a carbon sink in soil carbon cycling, especially under climate warming. Warming leads to increased intracellular glycogen and PHA accumulations within PAOs, allowing them to store carbon as intracellular polymers (Fig. 3). This accumulation significantly boosts soil organic carbon (SOC), as demonstrated by the positive correlation between PAO functional diversity and SOC (SI Fig. S10). By sequestering carbon within microbial biomass, PAOs help stabilize carbon in the soil, contributing to long-term carbon storage. Their role in storing carbon internally enhances the ability of the ecosystem to act as a carbon sink, buffering against carbon losses and ensuring that more carbon is retained in the soil despite environmental changes.

Although PAOs contribute to heterotrophic respiration (R_h, Pearson $r = 0.60$) through the decomposition of organic matter (OM, Pearson $r = -0.78$), their carbon storage capacity outweighs the carbon released via respiration. The positive correlation between PAO functional diversity and R_h reflects this contribution to carbon release, yet their negative correlations with net ecosystem exchange (NEE) and ecosystem respiration (ER) suggest that overall carbon uptake is still reduced. Moreover, the enhanced biotic interactions within the PAO community further support their ability to retain carbon in the soil, making PAOs a key component of the soil carbon sink. Their functional diversity ensures that the carbon stored through microbial processes surpasses the carbon lost, positioning PAOs as critical contributors to carbon sequestration under climate warming. By contributing to both the release and retention of carbon, PAOs ensure that ecosystems continue to cycle carbon efficiently, supporting soil fertility and long-term carbon storage.

Overall, PAOs play a crucial role in soil carbon cycling, functioning as both decomposers and carbon storers. Their ability to accumulate carbon storage compounds, mediate organic matter decomposition, and contribute to SOC levels highlights their dual role in releasing and sequestering carbon. In the face of climate change, resilience and functional diversity of PAOs offer a buffering effect against ecosystem carbon loss, ensuring that soil ecosystems can retain carbon and maintain nutrient cycling processes under environmental stress. Understanding the role of PAOs in soil carbon dynamics is essential for predicting how ecosystems will respond to global changes and for developing strategies to enhance soil carbon sequestration in the future.

2.2 Role of PAOs in Soil Phosphorus Cycling

PAOs also play a pivotal role in soil phosphorus cycling, particularly under the influence of climate warming. In response to increased temperatures, our study has observed significant changes in enzymatic activity and phosphorus availability in the soil. Specifically, alkaline phosphatase (AP) activity, which plays a crucial role in the hydrolysis of organic phosphorus compounds into absorbable inorganic phosphate, is significantly enhanced and positively correlated to PAOs (SI Fig. S3 & Fig. S10). This increase in AP activity under warming conditions suggests that PAOs and other microbial communities are adapting to maximize phosphorus acquisition from organic sources, potentially compensating for the decreased availability of total phosphorus (TP) in the soil (SI Fig. S3).

The decrease in TP implies a reduced input or higher utilization rate of inorganic phosphorus in the soil. This shift could be a result of several factors, including enhanced plant uptake driven by increased metabolic activity under warmer conditions or greater microbial assimilation into biomass. Consequently, PAOs, known for their capacity to accumulate and store phosphorus in the form of polyP, become crucial in phosphorus dynamics. They not only sequester phosphorus during periods of surplus but also release it through intracellular processes when external supplies are limited, acting as a buffer to maintain phosphorus homeostasis in the soil ecosystem.

Overall, these results together suggest that PAOs play an essential role in regulating soil phosphorus cycling under climate warming by enhancing AP activity to compensate for

decreased total phosphorus, while their ability to store and release phosphorus allows them to buffer nutrient availability and maintain soil fertility.

Supplementary Note F: FACS and downstream 16S rRNA amplicon sequencing protocol

To enable separation, quantification, and sorting of PAOs at the single-cell scale from soil, we developed a comprehensive and sequential workflow, including 1) sample pre-treatment that involves cell detachment and cell separation, 2) cell fixation, 3) triple-staining procedure, 4) FACS instrumental settings, 5) FACS gating strategies, 6) flow-cytometry counting, 7) FACS sorting, and 8) 16S rRNA gene amplicon sequencing.

The details of the protocol are sequentially described as below and mostly extracted as previously described [25].

1. Pre-treatment

1.1 Cell detachments: To isolate the microbial fraction, 1 gram of fresh soil was suspended in 20 mL of detachment buffer containing 3 mM sodium pyrophosphate ($Na_4O_7P_2$), 0.5% Tween 20, and 0.35% polyvinylpyrrolidone in 1X phosphate-buffered saline (PBS) [26]. The 1X PBS consisted of 0.4M Na_2HPO_4/NaH_2PO_4 and 150 mM NaCl at pH 7.2. The soil slurry was shaken for 30 min at room temperature in accordance with Deng et. al. [27], at 200 rpm using an orbital shaker to facilitate detergent based cell detachment.

1.2 Cell separation: Following cell detachment on orbital shaker, to enhance cell recovery, the resulting soil slurry was vortexed for 10 seconds and left to settle for 1 minute sediment large soil particles. Following sedimentation, cell separation from the soil matrix was further enhanced by decanting 10 mL of the soil slurry through a 40 μm cell strainer (Corning®, USA, Cat. No. 431750). The detached and filtered cell suspension remaining in the soil slurry were then pelleted through a 10-minute centrifuge at 6000g and 4°C. The soil microbe pellets were then washed three times with 1X PBS with centrifugation at 6000g for 10 minutes at 4°C.

2. Cell fixation: After the pre-treatment steps, the soil microbe pellets obtained from the previous step were then resuspended and fixed in 5 mL of fixation buffer at pH 7.2 for 24 hours at 4°C. The fixation buffer was prepared in accordance to previously published protocols [28, 29], containing 5 mM $NiCl_2$, 5 mM $BaCl_2$, 10% NaN_3, and 25 mM 4-(2-hydroxyethyl)-1-piperazineethanesulfonic acid (HEPES). The fixed soil microbe samples were then washed three times with 1X PBS with centrifugation at 6000g for 10 mins at 4°C, and resuspended into 1 mL 1X PBS awaiting further treatment for fluorescence staining. Prior studies have shown that fixed

microbial communities remain stable for up to 9 days, suggesting that fluorescence staining should be performed within this time frame to preserve community integrity [29].

3. Triple-staining procedure: After pre-treatment and cell fixation steps, the 1 mL cell suspension was then combined with 584 µL of Solution A (0.11 M citric acid and 4.1 mM Tween 20) and incubated for 20 minutes at room temperature. Then, the cells were further washed three times with 1X PBS and finally resuspended in 5 mL of 1X PBS before fluorescence staining.

Three fluorochromes were used to stain distinct cellular components, including SYBR Green I (Thermo Fisher Scientific, USA) for DNA [27], tetracycline hydrochloride (Sigma Chemical Co., USA) for polyP [28], and Nile red (Sigma Chemical Co., USA) for PHA [30]. Tetracycline has been successfully applied to stain PolyP in environmental samples, where more details can be found in previous studies [25, 28, 31, 32]. To characterize the fluorescence profiles for downstream gating strategy setups, seven aliquots of 300 µL each were subsampled from the prepared samples, including 3 aliquots for single-staining (each with one fluorochrome), 3 aliquots for double-staining (each with a unique pair of two fluorochromes), and 1 aliquot for triple-staining (all three fluorochromes). Staining was performed according to the fluorochrome combinations assigned to each aliquot. Single-stained aliquots received one of the following: For polyP staining, 112 µL of the 2 mg/mL stock solution of tetracycline hydrochloride in milli-Q water was added to the corresponding aliquots and incubated for 10 min at room temperature in the dark [28]; For PHA staining, 5 µL of the Nile red stock solution (10 mg/mL in DMSO) was introduced to the corresponding aliquots and then incubated for 30 min at room temperature in the dark [30]; For DNA staining, 10 µL of the 100X SYBR Green I was introduced into the relevant samples [27]. As for double-stained aliquots, they received two fluorochromes in the combinations polyP + PHA, polyP + DNA, or PHA + DNA, with dyes added sequentially in the order of tetracycline (10 min of incubation), Nile Red (30 min of incubation), and SYBR Green I, and incubation performed after each addition as applicable. The triple-stained aliquot received all three dyes in the same order and volumes, following the same incubation conditions after each addition. Each aliquot was then diluted by 1X PBS to final volumes of 1 mL to reach final concentrations of 0.225 mM, 0.05 mg/L, and 1X for tetracycline hydrochloride, Nile red, and

SYBR Green I, respectively. After staining, all samples were incubated at 4 °C in the dark for no less than 4 hours.

4. FACS instrumental settings: the quantification and sorting of PAOs were conducted using 100-micron nozzle on a Fusion Aria III cell sorter (BD Biosciences, USA), equipped with a violet laser (405nm excitation) with 525 nm BP filter for tetracycline hydrochloride, blue laser (488nm excitation) with 530 nm bandpass (BP) filter for SYBR Green I, and a yellow-green laser (561nm excitation) with a 610 nm BP filter for Nile red [28, 33, 34]. Forward and side scatter were used to assess cell size, granularity, and to distinguish cells from electronic noise [28].

5. FACS gating strategy to ensure PAO sorting at single-cell level: To ensure only cells are identified, quantified, and sorted at the point of interrogation during FACS, three more steps were performed. Firstly, the acceptable particle size was constrained by the ratio of forward scattering height (FSC-H) to forward scattering area (FSC-A) using the forward scattering channel (FSC). The acceptable particle size was determined by pure-cultured single-cell suspension of known bacterial PAO, *Tetrasphaera elongata* (DSM No. 14184). Secondly, to distinguish cells from background noise and non-cell events, unstained and single-stained controls were used to define gating thresholds for DNA (SYBR Green I), polyP (tetracycline), and PHA (Nile Red) [35, 36]. Unstained negative control was investigated in the SYBR Green I channel to exclude debris and non-cell particles based on SYBR Green I fluorescence. Thirdly, a sequential gating strategy was performed to ensure only cells with positive DNA+PHA+PolyP were identified as PAOs. Then, gating boundaries for polyP+DNA and PHA+DNA were established using tetracycline hydrochloride+SYBR Green I-positive (polyP+DNA-containing) and Nile red+SYBR Green I-positive (PHA+DNA-containing) controls. Finally, the candidate PAOs were identified as cells positive of all of SYBR Green I, tetracycline hydrochloride, and Nile red signals, indicating the presence of intracellular polyP, PHA, and DNA. Ultimately, this triple-staining FACS detects and quantifies microorganisms containing DNA, polyP, and PHA, concurrently, and was validated as a robust method for quantifying PAOs, comparable to single-cell Raman spectroscopy (SCRS) (SI Fig. S12 & Supplementary Note D) [37, 38].

6. Flow-cytometric counting: after the FACS instrumental settings and single-cell gating strategy implementations, flow-cytometric counting of the relative abundance of PAOs was

achieved by quantifying cell numbers of PAOs and total cells. PAOs were quantified as the number of triple-positive evens with positive signals of SYBR Green I, tetracycline hydrochloride, and Nile red fluorescence. The cells were quantified as the number of total SYBR Green I-positive events.

7. FACS sorting: after the application of gating strategy to quantify PAOs, FACS continued to sort PAOs for downstream microbial community analysis. Cells exhibiting positive DNA, polyP, and PHA fluorescence signals were sorted at a rate below 1,500 events per second, with 30,000 - 100,000 single cells sorted per replicate to obtain sufficient gDNA for 16S rRNA amplicon sequencing and the sorted PAOs were stored at -80°C [39].

8. DNA extraction and amplicon sequencing: genomic DNA was extracted from sorted cells by boiling at 100°C for 10 minutes using an Instagene matrix (Bio-Rad Laboratories, USA). To analyze the taxonomic composition of bacterial PAOs, the V4 region of the 16S rRNA gene was amplified with 515F and 806R primers, which included Illumina adapters and dual indices in accordance with Kozich et. al. [40]. The paired-end reads were processed using Mothur [41] and clustered at 99% similarity, with operational taxonomic units (OTUs) identified using the Silva database v138 [42].

More information on the pre-treatment steps for FACS sorting protocol on other soil samples can be referred to Yuan et. al. [25].

Supplementary Tables

Sample	Transformation	Power-law KS Distance	Power-law alpha	Power-law P value
**CC**	log	0.140	19.297	0.034
**UC**	log	0.150	7.734	0.019
**CW**	log	0.154	10.789	0.012
**UW**	log	0.131	8.204	0.048

Table S1 | PAO networks are scale-free. The table shows the results of the power-law fitting for different treatment conditions, where the KS distance represents the Kolmogorov-Smirnov distance, and the alpha parameter indicates the exponent of the power-law distribution. The lower p values indicates that PAO networks follow power law distribution. Each treatment (CC, UC, CW, UW) was log-transformed for the analysis.

Metrics	Symbol	Meaning	Inference on PAO network	+/-	Under warming	Good signal?	Summary
**Complexity**							
Average clustering coefficient	C	The higher the value, the more clustered or interconnected the network is.	Indicates higher local connectivity and potentially more robust and stable PAO networks.	+	↑	Y	
Average degree	$< k >$	The higher the value, the more connections each taxon has on average.	Suggests a more connected network with increased interactions among PAOs.	+	↑	Y	
Centralization of degree	C_D	The higher the value, the more centralized the network is around a few key taxa with high degree.	A more centralized network suggests reliance on a few key taxa, which could be points of vulnerability.	+	↑	Y	
Positive cohesion	$Cohesion_+$	The higher the value, the stronger the positive cohesion within the network, inferring more cooperative behaviors.	Indicates more cooperative interactions within the PAO network.	+	↑	Y	Enhanced network complexity in specialist PAO network. becomes more interconnected, cooperative, and efficient.
Connectance	$Conn$	The higher the value, the more densely connected the network is.	Reflects a more interconnected PAO network, potentially enhancing overall functionality.	+	↑	Y	
Density	D	The higher the value, the more densely packed the network is with edges.	Suggests a higher level of interaction among taxa, leading to a more resilient network.	+	↑	Y	
Number of edges	L	The higher the value, the more edges in the network.	Indicates a higher number of **Inference** interactions or relationships within the PAO network.	+	↑	Y	
Positive edge ratio	L_+	The higher the value, the more positive connections in the network.	Suggests a network with more positive, cooperative interactions among PAOs.	+	↑	Y	
Efficiency	E	The higher the value, the more efficient the network is in terms of information or resource flow.	Reflects efficient resource or information transfer within the PAO network.	+	↑	Y	
Geodesic efficiency	E_g	The higher the value, the more efficient the network is at a global scale.	Indicates overall network efficiency, enhancing global functional capabilities of the PAO network.	+	↑	Y	
Harmonic geodesic distance	H_g	The higher the value, the less efficient the network is in terms of shortest paths.	Suggests inefficiencies in the network path lengths, potentially hindering rapid information or resource flow.	+	↑	Y	

Maximal degree	K_{max}	The higher the value, the greater the highest degree of any taxa in the network.	Indicates the presence of a highly connected taxa, potentially a keystone species within the PAO network.	+	↑	Y	
Maximal eigenvector	E_{max}	The higher the value, the greater the highest eigenvector centrality of any taxa in the network.	Suggests key taxa with significant influence over the network.	+	↑	Y	
Number of network hub	Hub	The higher the value, the more key nodes acting as central points of connectivity in the network.	Reflects the presence of key nodes acting as central points of connectivity in the network.	+	↑	Y	
Transitivity	T	The higher the value, the more likely taxa are to form triangles, indicating a more tightly knit network.	Indicates strong local clustering, suggesting robust local subnetworks within the PAO network.	+	↑	Y	
Maximal betweenness	B_{max}	The higher the value, the greater the highest betweenness centrality of any taxa in the network.	Suggests key taxa that act as critical bridges within the network, essential for connectivity.	+	↓	Y	Reduce the network reliance on individual key taxa, distributing interactions more evenly and mitigating potential bottlenecks
Maximal stress	S_{max}	The higher the value, the greater the highest stress centrality of any taxa in the network.	Indicates taxa that experience significant control over resource or information flow, essential for network stability.	+	↓	Y	
Modularity	M	The higher the value, the more modular the network is, indicating strong community structure.	Reflects distinct community structures within the PAO network, enhancing functional specialization and resilience.	+	↓	Y	
Number of modules	Com	The higher the value, the more community in the network.	Indicates the presence of multiple functional groups within the PAO network, enhancing resilience and redundancy.	+	↓	Y	
Nestedness	N	The level to which the species interacting with specialists are subsets of those interacting with generalists.	Reflects hierarchical organization within the network, indicating resource or information flow efficiency.	+	↓	Y	
Relative modularity	RM	The higher the value, the more modular the network is relative to other networks.	Indicates a higher degree of modular organization compared to other networks, suggesting specialization.	+	↓	Y	
Relative nestedness	RN	The higher the value, the more nested the network is relative to other networks.	The negative RN means the PAO network is not trophic nested, and the decrease means the network is becoming less trophic under warming.	(+,-)	↓	N	

Negative cohesion	$Cohesion_{-}$	The higher the value, the stronger the negative cohesion within the network, inferring less competitiveness.	Indicates more competitive interactions, potentially destabilizing the PAO network.	-	↓	**Y**	
**Stability**							
Robustness	R	The higher the value, the more robust the network is to disturbances.	Reflects the network ability to maintain functionality despite disturbances.	+	↑	**Y**	Increased network stability
Vulnerability	V	The higher the value, the more vulnerable the network is to attacks or failures.	The decrease of vulnerability means the PAO network becomes less vulnerable under warming, indicating increased resilience.	+	↓	**Y**	

Table S2 | PAO network metrics. This table presents the key network metrics used to evaluate the complexity and stability of the PAO networks. Complexity metrics indicate the proportion of possible connections that are present. Stability metrics reflect the network ability to maintain structure under perturbation and the susceptibility to node removal. Altogether, these metrics provide insights into how the PAO network responds to environmental changes such as climate warming, with complexity generally promoting stability under stress conditions.

Reference

1. Xu X, Niu S, Sherry RA, Zhou X, Zhou J, Luo Y. Interannual variability in responses of belowground net primary productivity (NPP) and NPP partitioning to long-term warming and clipping in a tallgrass prairie. *Glob. Change Biol.* 2012;**18**:1648-1656
2. Barberán A, Bates ST, Casamayor EO, Fierer N. Using network analysis to explore co-occurrence patterns in soil microbial communities. *ISME J.* 2012;**6**:343-351 https://doi.org/10.1038/ismej.2011.119
3. Ma B, Wang H, Dsouza M, Lou J, He Y, Dai Z *et al.* Geographic patterns of co-occurrence network topological features for soil microbiota at continental scale in eastern China. *ISME J.* 2016;**10**:1891-1901 https://doi.org/10.1038/ismej.2015.261
4. Allison SD, Treseder KK. Warming and drying suppress microbial activity and carbon cycling in boreal forest soils. *Glob. Change Biol.* 2008;**14**:2898-2909
5. Wu L, Zhang Y, Guo X, Ning D, Zhou X, Feng J *et al.* Reduction of microbial diversity in grassland soil is driven by long-term climate warming. *Nat. Microbiol.* 2022;**7**:1054-1062 https://doi.org/10.1038/s41564-022-01147-3
6. Tian Y, Shi C, Malo CU, Kwatcho Kengdo S, Heinzle J, Inselsbacher E *et al.* Long-term soil warming decreases microbial phosphorus utilization by increasing abiotic phosphorus sorption and phosphorus losses. *Nat. Commun.* 2023;**14**:864 https://doi.org/10.1038/s41467-023-36527-8
7. Meng C, Tian D, Zeng H, Li Z, Yi C, Niu S. Global soil acidification impacts on belowground processes. *Environmental Research Letters*. 2019;**14**:074003
8. Hunt HW, Elliott ET, Detling JK, Morgan JA, Chen DX. Responses of a C3 and a C4 perennial grass to elevated CO2 and temperature under different water regimes. *Glob. Change Biol.* 1996;**2**:35-47
9. Zhou J, Xue K, Xie J, Deng Y, Wu L, Cheng X *et al.* Microbial mediation of carbon-cycle feedbacks to climate warming. *Nat. Clim. Chang.* 2012;**2**:106-110 https://doi.org/10.1038/nclimate1331
10. Davies M, Ecroyd H, Robinson SA, French K. Stress in native grasses under ecologically relevant heat waves. *Plos one*. 2018;**13**:e0204906
11. Zhou J, Deng Y, Luo F, He Z, Tu Q, Zhi X. Functional molecular ecological networks. *mBio*. 2010;**1**:10.1128/mbio.00169-10 https://doi.org/10.1128/mbio.00169-10
12. Xiao N, Zhou A, Kempher ML, Zhou BY, Shi ZJ, Yuan M *et al.* Disentangling direct from indirect relationships in association networks. *Proc. Natl. Acad. Sci. U.S.A.* 2022;**119**:e2109995119 https://doi.org/10.1073/pnas.2109995119
13. Deng Y, Jiang Y-H, Yang Y, He Z, Luo F, Zhou J. Molecular ecological network analyses. *BMC Bioinformatics*. 2012;**13**:113 https://doi.org/10.1186/1471-2105-13-113
14. Maslov S, Sneppen K. Specificity and stability in topology of protein networks. *Science*. 2002;**296**:910-913
15. Holloway LN, Dunn OJ. The robustness of hotelling's T 2. *Journal of the American Statistical Association*. 1967;**62**:124-136
16. van Iterson M, Boer JM, Menezes RX. Filtering, FDR and power. *BMC Bioinformatics*. 2010;**11**:1-11

17. Que X, Checconi F, Petrini F, Gunnels JA Scalable community detection with the louvain algorithm. IEEE. 28-37.

18. Yuan MM, Guo X, Wu L, Zhang Y, Xiao N, Ning D *et al.* Climate warming enhances microbial network complexity and stability. *Nat. Clim. Chang.* 2021;**11**:343-348 https://doi.org/10.1038/s41558-021-00989-9

19. Shi S, Nuccio EE, Shi ZJ, He Z, Zhou J, Firestone MK. The interconnected rhizosphere: High network complexity dominates rhizosphere assemblages. *Ecol. Lett.* 2016;**19**:926-936 https://doi.org/10.1111/ele.12630

20. Banerjee S, Schlaeppi K, van der Heijden MGA. Keystone taxa as drivers of microbiome structure and functioning. *Nat. Rev. Microbiol.* 2018;**16**:567-576 https://doi.org/10.1038/s41579-018-0024-1

21. Wang D, He P, Wang Z, Li G, Majed N, Gu AZ. Advances in single cell Raman spectroscopy technologies for biological and environmental applications. *Curr. Opin. Biotechnol.* 2020;**64**:218-229 https://doi.org/10.1016/j.copbio.2020.06.011

22. Li Y, Cope HA, Rahman SM, Li G, Nielsen PH, Elfick A *et al.* Toward better understanding of EBPR systems via linking Raman-based phenotypic profiling with phylogenetic diversity. *Environ. Sci. Technol.* 2018;**52**:8596-8606

23. Gu Q, Li Z, Han J. Generalized fisher score for feature selection. *arXiv preprint arXiv:1202.3725*. 2012

24. Li G, Wu C, Wang D, Srinivasan V, Kaeli DR, Dy JG *et al.* Machine learning-based determination of sampling depth for complex environmental systems: case study with single-cell Raman spectroscopy data in EBPR systems. *Environ. Sci. Technol.* 2022;**56**:13473-13484 https://doi.org/10.1021/acs.est.1c08768

25. Yan Y, Lee J, Baldwin M, Li G, Han IL, Wang Z *et al.* Discovering polyphosphate and polyhydroxyalkanoate-accumulating organisms across ecosystems: phenotype-targeted genotyping via FACS-sequencing. *bioRxiv*. 2025:2025-03 https://doi.org/10.1101/2025.03.24.644850

26. Eichorst SA, Strasser F, Woyke T, Schintlmeister A, Wagner M, Woebken D. Advancements in the application of NanoSIMS and Raman microspectroscopy to investigate the activity of microbial cells in soils. *FEMS Microbiol. Ecol.* 2015;**91**:fiv106

27. Deng L, Fiskal A, Han X, Dubois N, Bernasconi SM, Lever MA. Improving the accuracy of flow cytometric quantification of microbial populations in sediments: importance of cell staining procedures. *Front. Microbiol.* 2019;**10**:720

28. Gunther S, Trutnau M, Kleinsteuber S, Hause G, Bley T, Roske I *et al.* Dynamics of polyphosphate-accumulating bacteria in wastewater treatment plant microbial communities detected via DAPI (4′, 6′-diamidino-2-phenylindole) and tetracycline labeling. *Appl. Environ. Microbiol.* 2009;**75**:2111-2121

29. Günther S, Hübschmann T, Rudolf M, Eschenhagen M, Röske I, Harms H *et al.* Fixation procedures for flow cytometric analysis of environmental bacteria. *J. Microbiol. Methods*. 2008;**75**:127-134

30. Zuriani R, Vigneswari S, Azizan MNM, Majid MIA, Amirul AA. A high throughput Nile red fluorescence method for rapid quantification of intracellular bacterial polyhydroxyalkanoates. *Biotechnology and bioprocess engineering*. 2013;**18**:472-478

31. Mehlig L, Petzold M, Heder C, Günther S, Müller S, Eschenhagen M *et al.* Biodiversity of polyphosphate accumulating bacteria in eight WWTPs with different modes of operation. *J. Environ. Eng.* 2013;**139**:1089-1098
32. Günther S, Koch C, Hübschmann T, Röske I, Müller RA, Bley T *et al.* Correlation of community dynamics and process parameters as a tool for the prediction of the stability of wastewater treatment. *Environ. Sci. Technol.* 2012;**46**:84-92
33. Cao J-S, Xu R-Z, Luo J-Y, Feng Q, Fang F. Rapid quantification of intracellular polyhydroxyalkanoates via fluorescence techniques: A critical review. *Bioresour. Technol.* 2022;**350**:126906
34. Kawaharasaki M, Manome A, Kanagawa T, Nakamura K. Flow cytometric sorting and RFLP analysis of phosphate accumulating bacteria in an enhanced biological phosphorus removal system. *Water Science and Technology*. 2002;**46**:139-144
35. Li C, Zeng W, Li N, Guo Y, Peng Y. Population structure and morphotype analysis of “Candidatus Accumulibacter” using fluorescence in situ hybridization-staining-flow cytometry. *Appl. Environ. Microbiol.* 2019;**85**:e02943-18
36. Mori F, Nishimura T, Wakamatsu T, Terada T, Morono Y. Simple in-liquid staining of microbial cells for flow cytometry quantification of the microbial population in marine subseafloor sediments. *Microbes Environ.* 2021;**36**:ME21031
37. Majed N, Gu AZ. Application of Raman microscopy for simultaneous and quantitative evaluation of multiple intracellular polymers dynamics functionally relevant to enhanced biological phosphorus removal processes. *Environ. Sci. Technol.* 2010;**44**:8601-8608 https://doi.org/10.1021/es1016526
38. Majed N, Matthäus C, Diem M, Gu AZ. Evaluation of intracellular polyphosphate dynamics in enhanced biological phosphorus removal process using Raman microscopy. *Environ. Sci. Technol.* 2009;**43**:5436-5442 https://doi.org/10.1021/es900251n
39. Metz S, Lopes dos Santos A, Berman MC, Bigeard E, Licursi M, Not F *et al.* Diversity of photosynthetic picoeukaryotes in eutrophic shallow lakes as assessed by combining flow cytometry cell-sorting and high throughput sequencing. *FEMS Microbiol. Ecol.* 2019;**95**:fiz038
40. Kozich JJ, Westcott SL, Baxter NT, Highlander SK, Schloss PD. Development of a dual-index sequencing strategy and curation pipeline for analyzing amplicon sequence data on the MiSeq Illumina sequencing platform. *Appl. Environ. Microbiol.* 2013;**79**:5112-5120
41. Schloss PD, Westcott SL, Ryabin T, Hall JR, Hartmann M, Hollister EB *et al.* Introducing mothur: open-source, platform-independent, community-supported software for describing and comparing microbial communities. *Appl. Environ. Microbiol.* 2009;**75**:7537-7541 https://doi.org/10.1128/AEM.01541-09
42. Quast C, Pruesse E, Yilmaz P, Gerken J, Schweer T, Yarza P *et al.* The SILVA ribosomal RNA gene database project: improved data processing and web-based tools. *Nucleic Acids Res.* 2012;**41**:D590-D596
